# A new method of mark detection for software-based optical mark recognition

**DOI:** 10.1371/journal.pone.0206420

**Published:** 2018-11-09

**Authors:** Seng Cheong Loke, Khairul A. Kasmiran, Sharifah A. Haron

**Affiliations:** 1 Faculty of Medicine and Health Sciences, University of Auckland, Auckland, New Zealand; 2 Malaysian Research Institute on Ageing (MyAgeing), Universiti Putra Malaysia, Serdang, Malaysia; 3 Faculty of Computer Science and Information Technology, Universiti Putra Malaysia, Serdang, Malaysia; 4 Faculty of Human Ecology, Universiti Putra Malaysia, Serdang, Malaysia; University of Braunschweig - Institute of Technology, GERMANY

## Abstract

Software optical mark recognition (SOMR) is the process whereby information entered on a survey form or questionnaire is converted using specialized software into a machine-readable format. SOMR normally requires input fields to be completely darkened, have no internal labels, or be filled with a soft pencil, otherwise mark detection will be inaccurate. Forms can also have print and scan artefacts that further increase the error rate. This article presents a new method of mark detection that improves over existing techniques based on pixel counting and simple thresholding. Its main advantage is that it can be used under a variety of conditions and yet maintain a high level of accuracy that is sufficient for scientific applications. Field testing shows no software misclassification in 5695 samples filled by trained personnel, and only two misclassifications in 6000 samples filled by untrained respondents. Sensitivity, specificity, and accuracy were 99.73%, 99.98%, and 99.94% respectively, even in the presence of print and scan artefacts, which was superior to other methods tested. A separate direct comparison for mark detection showed a sensitivity, specificity, and accuracy respectively of 99.7%, 100.0%, 100.0% (new method), 96.3%, 96.0%, 96.1% (pixel counting), and 99.9%, 99.8%, 99.8% (simple thresholding) on clean forms, and 100.0%, 99.1%, 99.3% (new method), 98.4%, 95.6%, 96.2% (pixel counting), 100.0%, 38.3%, 51.4% (simple thresholding) on forms with print artefacts. This method is designed for bubble and box fields, while other types such as handwriting fields require separate error control measures.

## Introduction

Optical mark recognition (OMR) is a process that scans paper forms to detect darkened marks at pre-determined positions. It has been used in some way or other for almost 80 years to convert paper data into a machine-readable format for analysis. The popularization of personal computers in the 1980s gave rise to software OMR (SOMR) which allows anyone with a laser printer and scanner to custom design and print their own forms, which can then be scanned and converted using dedicated software [1].

SOMR has several advantages over hardware-based OMR in that supply costs are low, requiring only ordinary paper and printer toner, while the computer and scanner can be used for other purposes when not processing forms. In contrast, hardware-based OMR requires a dedicated and expensive scanning machine, and forms need to be specially printed at a cost of £100 (USD$125) per 1000 pages, or more if drop-out colors are used [2]. Scan speeds are about 10 pages per minute, which is lower than hardware OMR by an order of magnitude, but normally adequate for small-scale use.

Input fields in SOMR are typically bubbles or boxes which can be filled in with a soft pencil. These fields then need to be located on the scanned form before they can be detected and analyzed. Pre-printed registration marks enable field locations to be determined more accurately, thus allowing a greater density of input fields. Pattern recognition can be used to extract the fields, which are then converted to bitonal images, and marks determined by screening those above a minimum pixel count.

Bubble input fields are typically used with identification labels that are either external or internal to the bubble. Internal labels allow input fields to be closely spaced and appear neater but interfere with the pattern recognition routine required to locate the fields and detect user marks within the fields. In recent literature, there has been much written about new mark detection techniques such as adaptive X-mark matching, projection profiles, contrasting classifiers, finder patterns, and simple thresholding [3–7]. None of these methods however accept internal labels.

Adaptive X-mark matching utilizes template matching to detect cross marks made in rectangular box fields. Template matching is the process whereby a template image can be located within a larger image by sampling each point within the larger image and comparing the strength of the match. While accuracy is high and detection of colored pen marks is possible, the boxes need to have a minimum size of 0.8 x 0.8 cm. Moreover, rotated marks cannot be consistently detected (57.2% error rate) unless shifted and/or orientated templates are used (0.02% error rate), which greatly adds to the computational complexity of the method [3].

The projection profile method once again uses rectangular boxes, but compares the marks made within a single form to determine a threshold for classification using a histogram of pixel counts. This has the advantage of accounting for variability in filling out input fields between respondents, but has a higher error rate (0.09%) and requires a fixed grid of boxes to be effective [4].

The study on contrasting classifiers compared four types of classifiers and found that only simple pixel counting was accurate enough to be used (error rate 0.02%). Pixel counting is the classification of a mark based on the fraction of scanned pixels in an input field which exceed a pre-determined threshold. However, only completely darkened bubbles with soft pencils could be detected consistently by this method. The other classifiers had more flexibility but suffered higher error rates ranging from 3.95 to 26.43% [5].

The finder pattern method uses a large rectangular box at the print borders to accurately remove any rotation in the scanned image. The input fields are then extracted based on calculated positions and simple pixel counting employed to detect the marks. Accuracy is the same as for the contrasting classifiers as they use the same underlying method for detection, but suffers similar drawbacks [6].

Simple thresholding is like the projection profile method but detects darkened bubbles instead of cross marks in rectangular boxes. The error rate is however much higher (0.80%) as erased bubbles are erroneously detected more often than erased cross marks [7].

In general, existing methods for mark detection perform adequately when respondents use soft pencils, completely darken bubble fields, or consistently use a fixed mark shape. These constraints are acceptable when forms are used in a supervised setting, such as for student examinations or when filled by trained personnel, as adherence to instructions is incentivized. When used for self-completed community surveys, respondents are untrained and typically use whatever writing instruments are on hand, such as hard pencils or colored ball-point pens. Rather than completely darkened bubbles, tick marks or crosses are often found (Table 1). The printing and scanning process can also leave visible artefacts on the form, especially when the equipment is old, heavily utilized, or in need of servicing. These artefacts are marks on the scanned version of the form which are not present in the original, and usually arise from hardware or software errors during the digitization process.

**Table 1:** A comparison of published error rates between different SOMR methods used in recent studies with the new detection method from this study.

	Projection Profile [4]	X-Mark Detection [3]	Pixel Counting [5]	Simple Thresholding [7]	New Method
**Error Rate (%)**	0.09	0.02	0.02	0.80	**0.03% (untrained)** **0.00% (trained)**
**Sample Size**	16500	6400	11640	5000	**6000 (untrained)** **5695 (trained)**
**Tested on Students**	X		X	X	
**Tested on Members of Public**		X			**X**
**Bubbles Not Completely Darkened**	X	X			**X**
**Internal Labels**					**X**
**Able to Cope with Different Writing Instruments**		X			**X**
**Comments**		X-marks only	Best of four methods		

Note: The published error rates shown in the table are defined as the number of detection errors divided by the number of input fields (sample size) during testing.

The faint tick marks from a hard pencil give the software fewer pixels to detect, and digital subtraction of internal labels further invalidates a proportion of these pixels. Print and scan artefacts add noise to the image, thus making it even harder to correctly determine marks. This article discusses a new method of detection in SOMR which can accurately classify marks even when all these factors are present.

## Mark detection

This new detection method was developed in conjunction with a new forms processing application which implements SOMR [8]. It was found during preliminary field testing that the standard methods of mark detection were not accurate enough when used on forms filled by untrained respondents. The equipment used for printing and scanning were heavily used, resulting in many scanned forms having artefacts of some sort. Finally, there was a need for internal labels for bubble input fields as the bulk of our survey forms used these fields due to the higher SOMR accuracy compared to handwriting fields. Questions with internal labels were typically 30–50% more compact than those with external labels, allowing us to print forms with fewer pages.

This detection method consists of three sequential processes, which are removal of internal labels, basic mark detection, and correction of print and scan artefacts. The removal of internal labels is done by masking out the labels with a template, so that any residual pixels can be attributed solely to user marks. Mark detection involves simple pixel counting after pre-processing to remove noise. Finally, common print and scan artefacts are corrected for using a statistical technique that isolates user marks from the artefacts.

### Removal of internal labels

Prior to printing, the exact position of all bubble input fields and the content of internal labels is first stored in a data file. After the forms have been filled, these are then scanned into image files, and have any rotation or displacement corrected. The key requirement for this process is pixel-accurate determination of bubble field locations in the scanned image.

**Fig 1:**
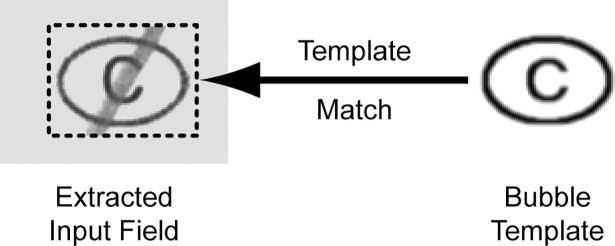
Extraction of the input field and determination of its exact location using template matching.

The first step is extraction of the scanned input field together with a margin of a few pixels to account for small inaccuracies in the realignment process. An image of the bubble together with the internal label is generated, and used to determine the location of the scanned bubble through standard cross-correlation template matching (Fig 1).

Cross-correlation detection generates a map array where areas in the source image with the highest similarity to the template will be represented as peaks. Thresholding of the map array will give blobs, and determination of the centroids using image moments will give the position of the input field with sub-pixel accuracy. This process however fails if the entire bubble is darkened, and the input field is then extracted based on the location stored in the data file.

**Fig 2:**
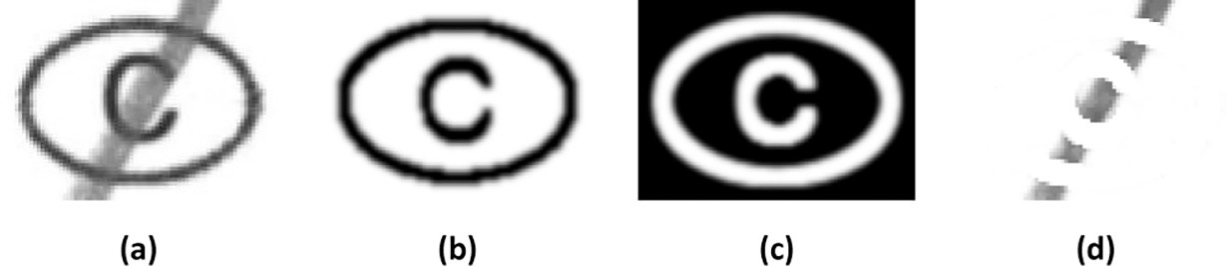
Steps in removal of the bubble-label template. Note: (a) The scanned input field extracted with a narrow margin. (b) The bubble-label template. (c) Inversion and morphological dilatation. (d) Template overlaid on the scanned input field.

**Fig 3:**
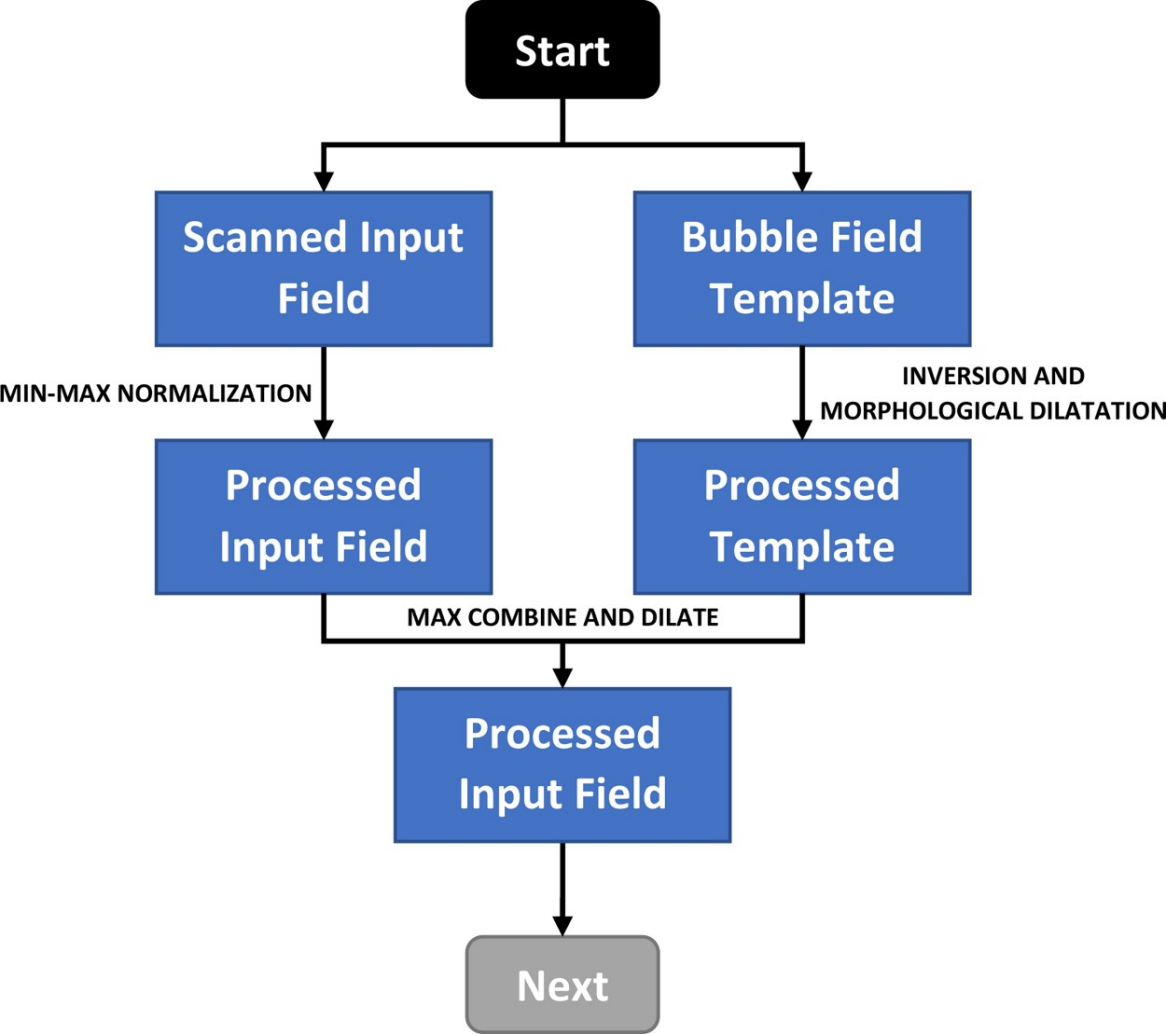
Flow diagram for removal of the bubble-label template. Note: “Start” refers to the beginning of the algorithm where the inputs consist of a scanned input field and the corresponding bubble field template. “Next” refers to the following section in Fig 5.

The second step involves inversion and morphological dilatation of the bubble-label template using a small kernel structuring element, to give a template with white areas that have been expanded by a single pixel. This is then overlaid on the scanned bubble field to leave just the user marks in the image (Fig 2 and Fig 3). Without pixel-accurate location of the input field, the above masking process will leave pixel residues which can interfere with mark detection.

The structuring element is a pre-defined shape that is used to interact with the underlying image through a morphological operation such as a convolution filter. A convolution filter processes an image by multiplying each pixel with a matrix kernel in the form of a structuring element.

### Basic mark detection

Bubbles which have been completely darkened are easy to detect, requiring just binary thresholding and selecting marks with a pixel count above a pre-set proportion. Ticks and crosses are more problematic in that the pixel intensity usually varies along the path of the stroke. To account for this variation, Gaussian adaptive thresholding is applied based on a neighborhood block size of 11 pixels at 300dpi, which is the approximate width of a typical mark drawn with a soft pencil. Adaptive thresholding is a technique that sets the threshold for binarization based on the Gaussian average of pixels in the neighborhood. This contrasts with normal binary thresholding that sets the threshold based on a pre-determined value or from the global average of the image.

**Fig 4:**
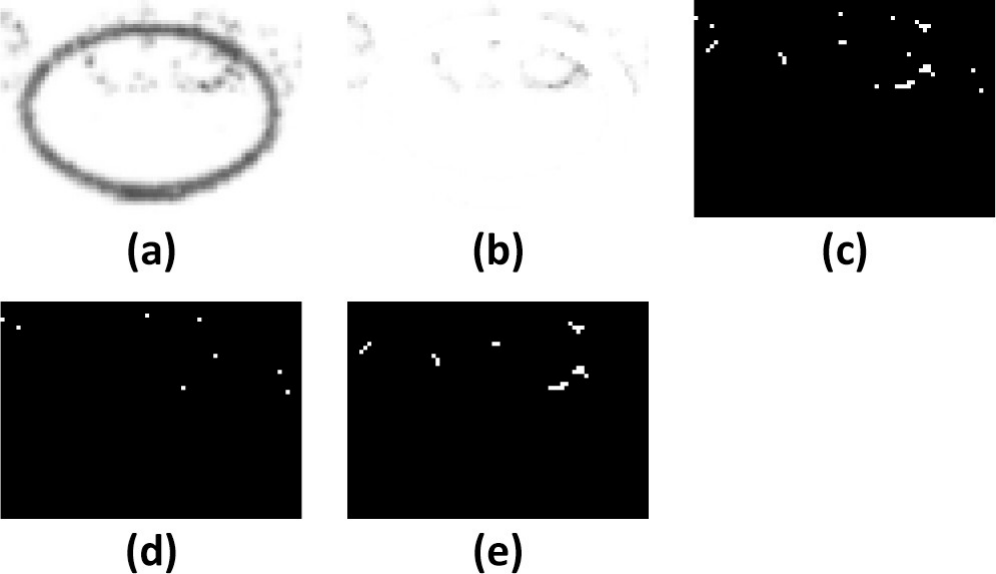
Stages in removal of "salt and pepper" noise. Note: a) Original scanned bubble with a band of “salt and pepper” deposits on the top half. b) Extracted markings. c) Gaussian adaptive thresholding. d) Isolated dot extraction using a convolution filter. e) Markings with isolated dots removed.

**Fig 5:**
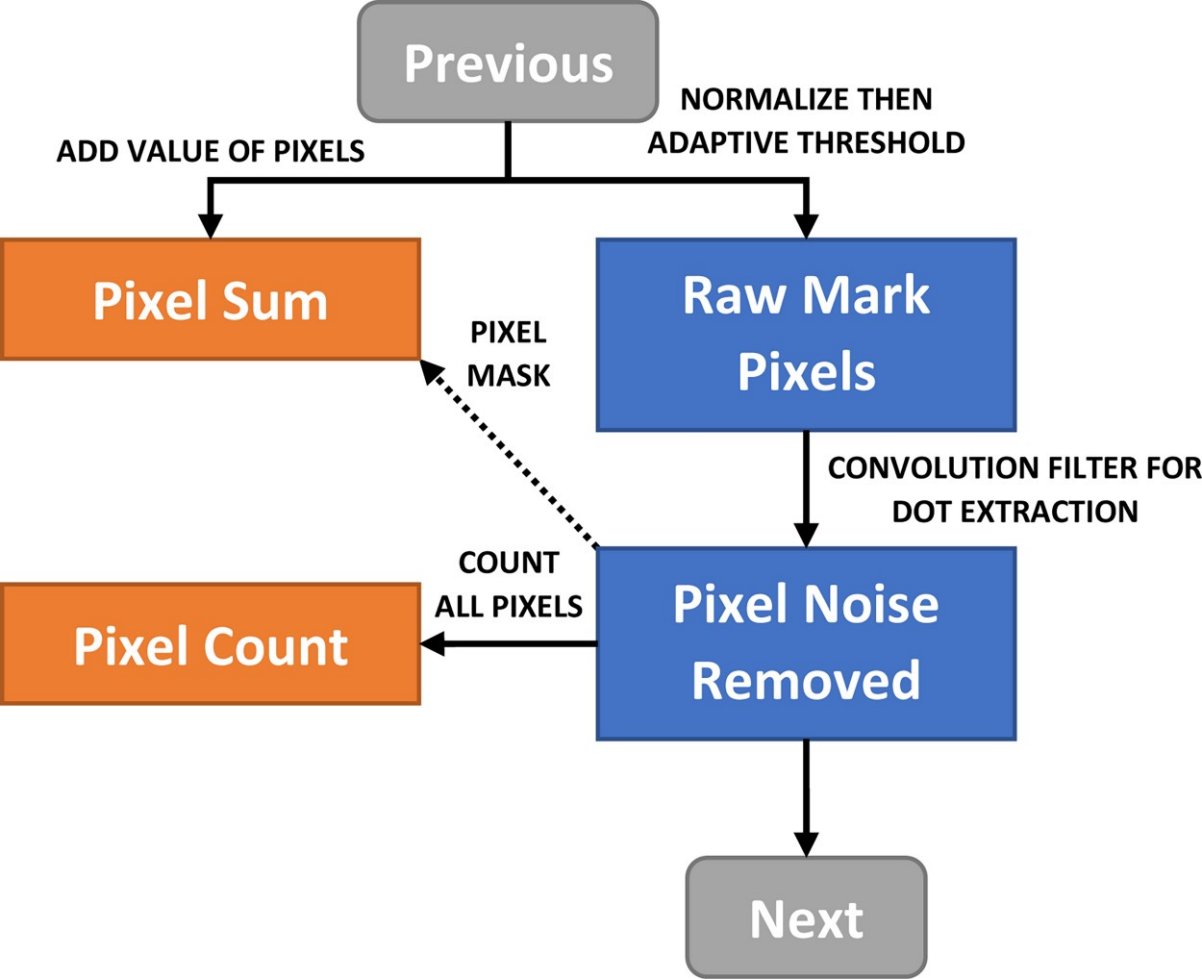
Flow diagram for removal of "salt and pepper" noise. Note: “Previous” refers to the preceding section in Fig 3. “Next” refers to the following section in Fig 9.

The threshold is set 56 units below the Gaussian weighted mean of the pixels in the neighborhood block for 8-bit greyscale pixels, which is the mean pixel value weighted using a Gaussian function. This enables even the faint tail of the stroke to be detected, which otherwise would be removed in normal binary thresholding (Fig 4 and Fig 5). The threshold was obtained empirically by adjusting the value until the faint tail could be seen for most marks, while minimizing pixel artefacts.

### Print and scan artefacts

There are several types of artefacts which are commonly found in scanned forms. The first type consists of “salt and pepper” deposits which occur when the laser printer drum is dirty, the fusing mechanism’s temperature is incorrectly set, or double-sided printing is used. These deposits typically appear as pixel noise on the scanned image and are best removed using a small kernel convolution filter (3x3 structuring element at 300dpi) which is better at removing noise than a simple median filter (Fig 4).

If the level of noise is very heavy, it is not possible to reliably classify marks just using morphological transformations. If the structuring element is too small, clusters of pixels will remain, and if the element is too large, it will remove marks which are faint. In the example given, isolated pixels are easily removed, leaving several clusters behind (Fig 4).

**Fig 6:**
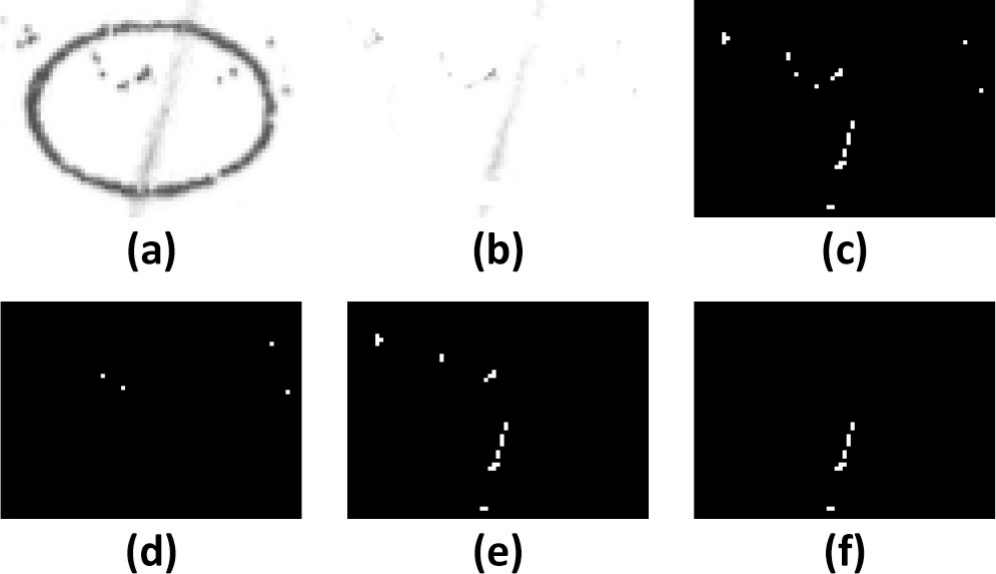
RANSAC filter to isolate linear strokes. Note: a) Original scanned bubble with a band of “salt and pepper” deposits on the top half and a faint pencil stroke. b-e) Removal of “salt and pepper noise”. f) Image filtered using RANSAC to isolate pixels from the linear stroke. In the normal processing pathway, the faint pencil mark would not be detected because of the noise in the image.

**Fig 7:**
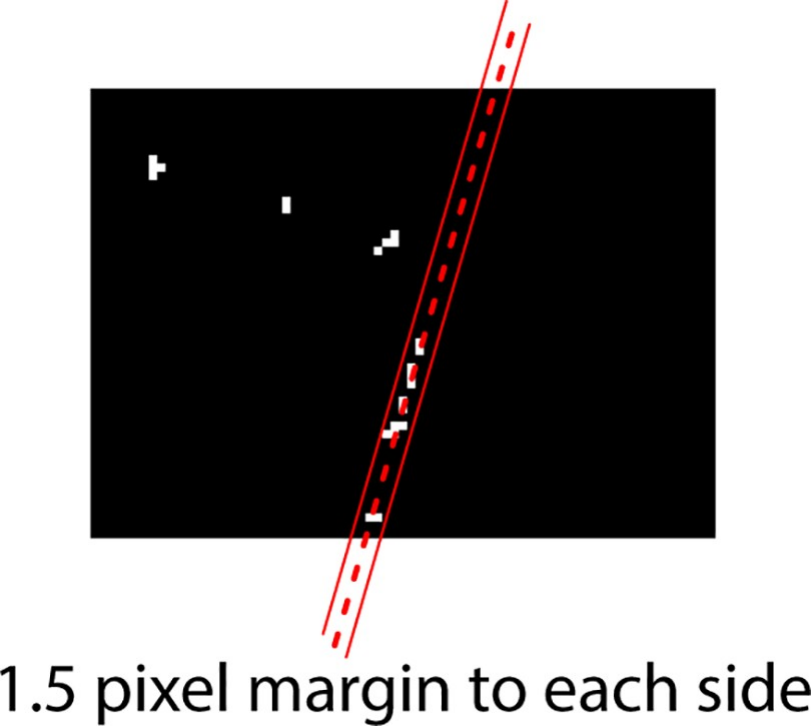
RANSAC filter showing the isolated line with a margin of 1.5 pixels on either side.

One way around this is to filter the remaining pixels and identify those grouped in a straight line using a random sample consensus (RANSAC) technique, which is a statistical method used to isolate outliers [9]. If the proportion of linear pixels compared to the total detected pixels exceeds a threshold, this then counts as a detected mark (Fig 6). The margin for identifying line pixels should be set to about 2% of the diagonal length of the bubble. For example, a bubble with a diagonal of 80 pixels can have a margin of 1.5 pixels to either side of the line (Fig 7).

**Fig 8:**
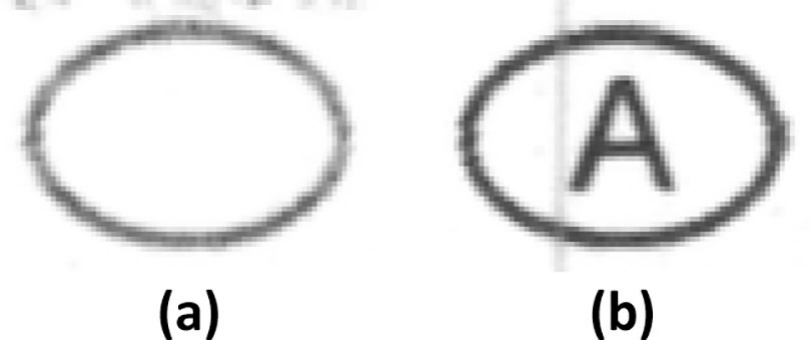
Examples of linear artefacts. Note: Examples of linear artefacts which can mimic strokes on the RANSAC filter. In (a), there is a horizontal band artefact above the bubble, while (b) has a vertical black line running through the bubble.

Another type of print artefact would be horizontal or vertical linear lines (Fig 8). These occur due to defects or wearing out of the toner cartridge or fuser in the laser printer [10]. User-entered marks are almost always at an angle to the axes. Hence, linear artefacts can be detected by estimating the slope of the line on the RANSAC filter and removing those that are vertical or horizontal. Once these artefacts are removed, another iteration of the detection routine can be run to detect actual marks.

In general, when the pixel count is very high, this always indicates that a mark is present. It is only when the count is moderate that there is a possibility of misclassification in the presence of noise. For efficient processing, an upper limit can be set above which a mark is considered detected. For pixel counts between 33–100% of this limit, the RANSAC filter can be applied to differentiate marks. Pixel counts below 33% are always considered as no mark present.

**Fig 9:**
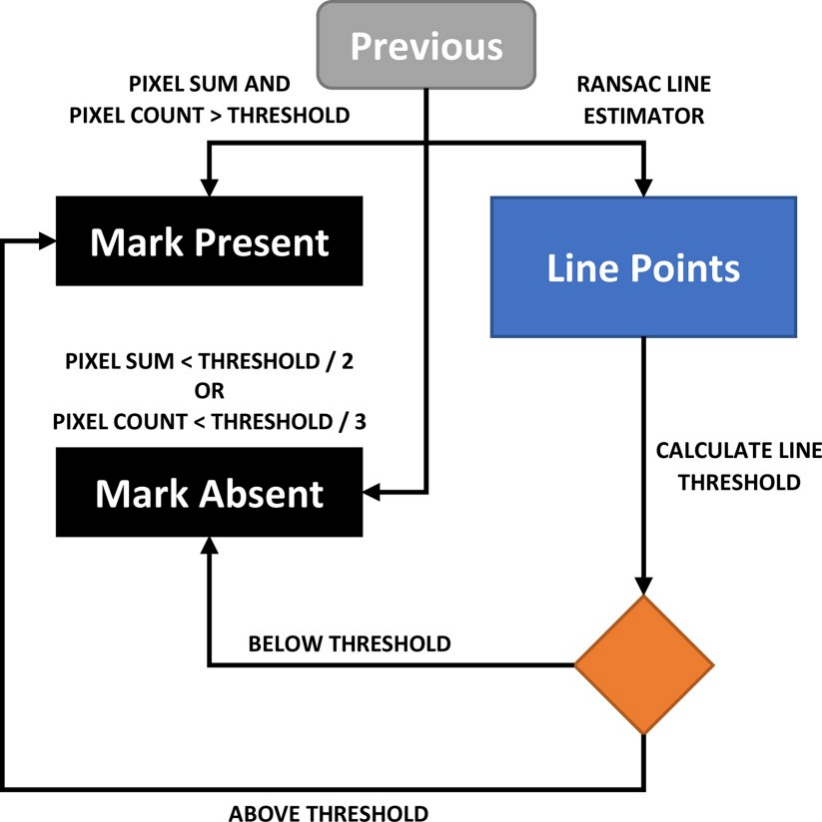
Flow diagram for RANSAC filter implementation. Note: “Previous” refers to the preceding section in Fig 5. The end points of the algorithm are a binary decision on whether a mark is present or not.

Another parameter that can be used to differentiate marks is the pixel sum. In contrast to the pixel count, the pixel sum is the additive sum of the original values for all pixels isolated after adaptive thresholding. This parameter is weighted towards detecting lighter pixels and will pick up fainter marks (Fig 9). In the same way, pixel sums between 50–100% of the upper limit are processed using the RANSAC filter. These thresholds were determined empirically from an informal sample (25 forms each containing 25 input fields) to optimize classification accuracy, and using both the pixel count and pixel sum will detect more marks than either parameter alone.

**Fig 10:**
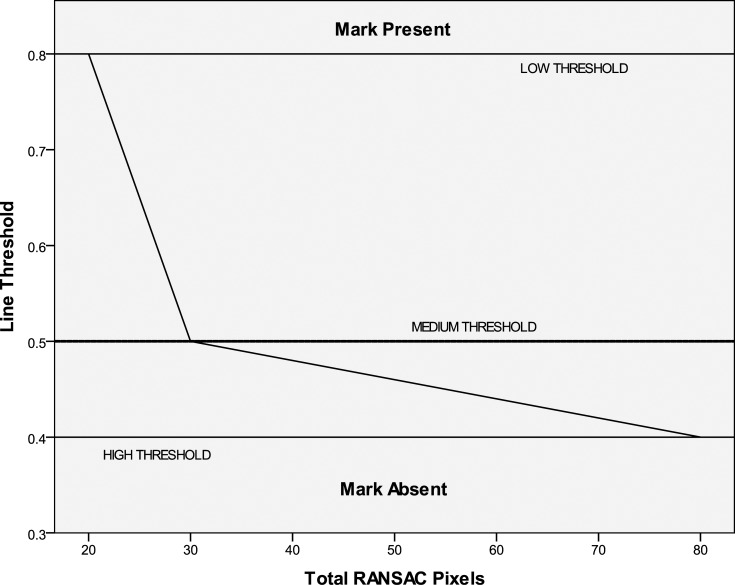
Calculation of line threshold.

When using the RANSAC filter, determining the threshold proportion of linear pixels compared to the total detected pixels is tricky. It was found that when a small number of detected pixels were present, this threshold needed to be set higher as noise would constitute a bigger proportion of the pixels. In contrast, when there were more detected pixels, the threshold should be set lower to detect multiple lines such as for a cross mark (Fig 10). The source image for the RANSAC filter was also processed using Gaussian adaptive thresholding with the method described in Fig 4 and Fig 5, but with a threshold level set to extract lighter pixels. In this case, the threshold is set 8 units below the Gaussian weighted mean of the pixels in the neighborhood block, for 8-bit greyscale pixels. Once again, this threshold was obtained empirically by adjusting the value until most marks could be fully visualized, regardless of pixel artefacts.

### Example using the RANSAC filter

For the bubble shown in Fig 2, the maximum pixel sum and pixel count can be determined using a completely darkened input field together with the margins (Fig 5). At 300dpi 8-bit greyscale, the maximum pixel sum is 727033, while the maximum pixel count is 427 pixels.

Based on empirical testing with the 43 input fields containing visible print artefacts (Table 2), the pixel sum threshold should be fixed at 0.35% of the maximum pixel sum, which in the above example is set at 2500. The pixel count threshold is set to 10% of the maximum pixel count, which in the above case is 45 pixels.

**Table 2:** A comparison of error rates for bubble input fields from the three surveys.

Survey Filled By:	Investigator		Enumerator		Respondent	
	N	%	N	%	N	%
**User errors**	0		0		10	0.167
**Print and scan artefacts**	0		0		7	0.117
**Software misclassification**	0		0		2	0.033
**Total field count**	2295		3400		6000	

Note: For purposes of clarity, the percentage error rate is given only for non-zero rates. Single-selection fields are counted as a single entry each, while multi-selection fields are counted as separate entries. For the enumerator-administered survey, 21 fields had detectable artefacts, while the respondent-administered survey had 22 fields with artefacts.

If both the pixel sum and pixel count are above these thresholds, then a mark is detected. If either the pixel sum or the pixel count are below 50% (1250) or 33% (15) of these thresholds respectively, then a mark is not detected. Otherwise, the RANSAC line estimator is used to classify the mark (Fig 9).

The input image for the line estimator is first re-processed to extract lighter pixels. The pixels are then run through the RANSAC routine and the proportion of linear pixels is then determined, ranging from 0–1.

The next step is to determine the low, medium, and high pixel count thresholds which are set to 5%, 7%, and 20% of the maximum pixel count respectively, or 21, 30, and 85 pixels based on the above example. The corresponding line thresholds for these pixel counts are fixed at 0.8, 0.5, and 0.4 respectively, and intervening values can be determined from the graph in Fig 10.

If the proportion of linear pixels from the RANSAC routine exceeds the line threshold for the corresponding pixel count, then a mark is detected. For example, 30 pixels were extracted after re-processing, and 20 of these were found to be linear after RANSAC, giving a proportion of 0.67. As this value is greater than the line threshold of 0.5 for the RANSAC pixel count of 30 (Fig 10), a mark is then detected.

An implementation of this algorithm can be found at https://github.com/scloke/Survey2/blob/master/Scanner/ in the function “DetectChoice” of the file Scanner.xaml.vb, and the parameter values given in the above example come directly from the algorithm when run on the bubble shown in Fig 2.

## Test results

### Field-testing of detection method

The new detection routine was field-tested using three sets of forms from two studies, the first of which was completed by one of the investigators, the second by an enumerator (person employed to conduct the survey) with basic training, while the third was filled by untrained members of the public. Detection errors were divided into three categories: user errors where there was a mistake in filling the field, errors arising from print and scan artefacts, and finally errors caused by misclassification from the detection routine.

The first study was the “Renal Hyperparathyroidism Study” conducted at Hospital Sultan Ismail from 2011–2015 with data from 85 subjects [11,12]. All forms were filled by the lead investigator using data from a spreadsheet, with subjects matched to identification numbers (S1 Fig). The second study was the “Health Perceptions Among the Malaysian Public” community survey, with forms designed in self-administered and interviewer-administered formats (S2 Fig) [13].

The survey forms for this study were constructed based on components sourced from previous large scale community surveys in Malaysia [14,15]. The self-administered survey form was tested on 75 subjects recruited from a public location, while the interviewer-administered form was tested on 50 subjects recruited from a housing estate and filled by a single enumerator. For these surveys, written informed consent was obtained from each subject based on a study information leaflet. The period of data collection was the month of October 2016 and was based on convenience sampling as this was not expected to cause any material bias since only the marks were being assessed rather than the content of the surveys. No information was recorded by the enumerator on the refusal rate for respondents. The sole inclusion criterion for recruitment was that the respondents were able to read and complete the forms. Data on error rates for the non-bubble fields from field-testing is available in an accompanying publication (Tables 2 and 3 in S3 Fig).

Double-keying was used to ensure accuracy for the final data set. This was achieved by comparing the software detected marks with those entered separately by a single data entry clerk. All discrepancies were then resolved by the lead investigator and the types of error were noted.

The first study was granted full ethics approval by the Malaysian Ministry of Health (KKM/NIHSEC/P15-617)(NMRR-15-554-24819 (IIR)), while the second study was granted exemption from review by the ethics board of Universiti Putra Malaysia (UPM/TNCPI/RMC/1.4.18.1(JKEUPM)/004).

From Table 2, the survey filled by untrained respondents had a high number of user input errors. When the forms were inspected, it could be seen that the writing instruments used and the way the bubbles were marked also varied greatly. Despite this, only 0.033% of the fields were misclassified by the detection routine, while the investigator and enumerator-filled surveys had no misclassification. The routine was also able to correctly classify 83.7% of the fields with print and scan artefacts.

**Fig 11:**
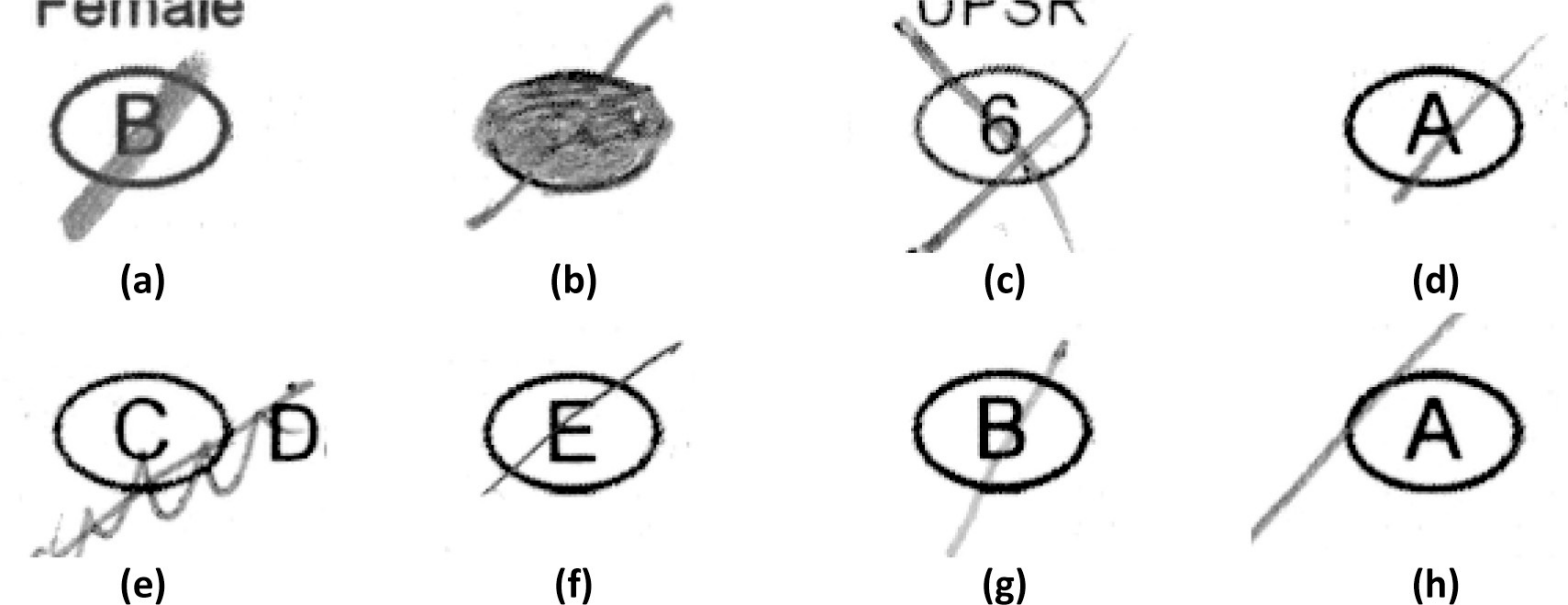
Examples of input fields filled by untrained responders.

In Fig 11, sample (a) shows a mark that is well filled with a tick and is easily detected by the new method and possibly by the projection profile method. Sample (b) is completely filled, and any detection routine should be able to detect this mark. Sample (c) is a cross mark, and only the X-mark, projection profile, and the new method can detect this. Sample (d) is tapered and without adaptive thresholding, the fainter portion of the mark will be lost on processing. Sample (e) is an example of a user error caused by cancellation rather than erasure. Sample (f) is filled using a hard pencil, which leaves a thin mark with fewer pixels to be detected. Sample (g) is filled using a colored pen, which gives a mark that has a lighter shade of grey. Sample (f) has a tick right at the edge of the bubble. The new method however can detect this mark as it also analyses the immediate area surrounding the bubble based on the extraction margin.

### Comparison with other detection methods

A direct comparison was made between the new method, pixel counting, and simple thresholding detection methods [5,7]. These were tested on bubble images isolated from the forms in the above two studies, where mark classifications were already known through a double-keying process.

The projection profile and X-mark detection methods were not tested as they can only be used on boxes with ‘X’ marks. In any case, this type of input field is commonly used only in Thailand for public and school examination papers [3,4].

During testing, it was found that the suggested cut-offs from the literature for the two methods needed to be recalibrated for the bubbles used in the forms. This was done using receiver operating characteristic curves to maximize the Youden's J statistic, which is a measure of “informedness” that takes into account all predictions [16]. The comparison was made based on the ability to detect marks on bubbles without artefacts, bubbles with artefacts, and the combined bubble sample.

It was found that the new method had the highest accuracy in the comparison (99.94%) and was especially effective in distinguishing marks for bubbles with artefacts. The simple thresholding method also performed well in bubbles without artefacts (99.80%), but the accuracy dropped substantially where artefacts were present (51.39%). The accuracy for the pixel counting method was poor (96.06%) in the sample without artefacts, but this did not seem to change even where artefacts were present (96.18%) (Table 3). The detection accuracy for individual bubbles with artefacts in Table 3 is higher than for fields in Table 2 as each field may contain multiple bubbles, and a detection error in any one of these invalidates the entire field.

**Table 3:** A comparison between the new method, the pixel counting, and simple thresholding detection methods based on a sample of bubbles with and without artefacts.

	Sensitivity (%)	Specificity (%)	Accuracy (%)	Youden’s J (%)
**No Artefact**				
New Method	99.72	100.00	99.95	99.72
Pixel Counting	96.26	96.01	96.06	92.28
Thresholding	99.86	99.79	99.80	99.65
**Artefact**				
New Method	100.00	99.12	99.31	99.12
Pixel Counting	98.36	95.59	96.18	93.96
Thresholding	100	38.33	51.39	38.33
**Combined**				
New Method	99.73	99.98	99.94	99.71
Pixel Counting	96.31	96.01	96.06	92.31
Thresholding	99.86	98.73	98.94	98.60

Note: The combined sample consisted of 15837 bubbles (2950 marked), 288 with artefacts (61 marked), and 15837 without artefacts (2889 marked). Cut-offs for the pixel counting and simple thresholding methods were set to maximize the Youden’s J statistic for the combined sample.

## Discussion

Recent studies using different implementations of SOMR have reported error rates between 0.02–0.80% [3–5,7,17–20]. The error rate using the new detection method was 0.03% when tested on untrained respondents, which is in the lower end of the range compared to the others. When tested on trained personnel, the error rate was essentially zero. The main difference from the other methods is that they require that either the bubbles be completely darkened, have no internal labels, or be clearly filled with a black pen or soft pencil (Table 1). The comparison between the reported error rates depends in part on the testing dataset, and it is noteworthy that the dataset for the new method used untrained respondents and internal labels, both of which incur a higher error rate than the datasets from the published studies [13].

In a clinical trial, acceptable error rates are less than 0.1% for critical data fields and less than 1% for non-critical fields [21]. The new detection method is accurate enough to fulfil these criteria under most circumstances. Even under unfavorable conditions with print artefacts and filled by untrained respondents, the error rate only rose to 0.15%.

When compared with the commercial RecoFlex recognition engine in Hewlett-Packard’s industry-standard TeleForm, which is a neural-based recognition system that combines multiple recognition engines for improved accuracy, the SOMR component yielded an error rate of 0.02% which is comparable to the new detection method described. Moreover, the stated accuracy for TeleForm required 153 out of 636 questionnaires to have the bubble markings darkened, amended, or marked over with a black pen when tested on untrained respondents [13,22,23]. Internal labels were also not used.

The low error rates for the “X-Mark Detection” and “Pixel Counting” methods were achieved only by looking for specific types of marks, which were crosses and completely filled bubbles respectively, while favorable testing conditions were created for TeleForm [3,5,22]. The new detection method can achieve similar results under a wide variety of conditions, which these other methods are not able to do. This is further reinforced by the direct comparison in Table 3, which demonstrated that not only is the baseline accuracy of the new method better, but its performance is maintained even in the presence of print and scan artefacts.

The new method can be applied to all implementations of SOMR, as it can be used for bubble as well as box input fields and is not dependent on a particular layout such as with the projection profile method. The minimum size of the input fields is smaller than for the other methods, being about 0.55 x 0.45 cm. This method is not restricted to a single mark design as with most of the other methods described in Table 1, and is tolerant of mark variation between respondents and different writing instruments. If pencil marks are properly erased and pen marks covered completely with correction fluid, the detection process is largely unaffected.

The method is also very fast, and when tested on a system using Microsoft Windows 10 with an Intel Xeon E3 processor running at 3.5 GHz (475 MFLOPS) using Visual Basic 2015, 700 input fields per second could be processed without RANSAC, and 300 fields per second with RANSAC. On average, only 1% of bubble input fields filled by trained personnel required RANSAC processing, compared with 2.4% of fields filled by untrained respondents.

The main shortcoming of the new method is that the various cut-offs need to be set for each specific bubble design, size, and scan resolution. However, this is easy to do as the maximum pixel sum and pixel count for a completely darkened bubble including the margins need to be first determined, then the various cut-offs can be obtained by scaling against the values provided in the example shown in Fig 10. The structuring elements for the morphological filters will also need to be scaled according to the scan resolution. For example, at a scan resolution of 600dpi, the elements will need to be twice the size of what was used in the above example. This should not be an issue in most implementations as bubble sizes are usually fixed for a particular SOMR package, and moreover the values can be determined automatically using the above routine.

Finally, it needs to be understood that while the improved method for mark detection can raise the accuracy for bubble and box fields, the error rate for handwriting fields remains at about 1% [8]. This is best handled at the form design stage where handwriting fields are kept to a minimum and restricted to non-critical sections. For critical sections where the use of these fields cannot be avoided, a double-keying process should be instituted to correct errors at the processing stage. The error rate for the improved method is low enough that double-keying is not needed even for critical fields unless the forms are very dirty or contaminated with print artefacts (Table 3) [21].

## Conclusion

The new method of mark detection in SOMR represents an improvement over existing methods employed in commercial software as well as others developed in recent years. Its main advantage is that it can be used under a variety of conditions and yet maintain a high level of accuracy that is sufficient even for scientific applications. While this method contributes to improving data integrity, care should also be made to reduce the number of handwriting fields and screen these with a double-keying process to correct remaining errors.

## Supporting information

S1 Fig Data collection form for the Renal Hyperparathyroidism Study. (PDF)

S2 Fig Data collection form for the Health Perceptions Among the Malaysian Public survey. (PDF)

S3 Fig Accompanying publication with data on error rates from field testing according to field type. (PDF)
