# Supplementary material for: A new method of mark detection for software-based optical mark recognition
Source: PLoS One. 2018 Nov 9;13(11):e0206420. doi: 10.1371/journal.pone.0206420 (PMC6226159; doi:10.1371/journal.pone.0206420)
Supplement: S1 Fig — (PDF) [file pone.0206420.s001.pdf]

Post-Parathyroidectomy Study Form

Section 1 - Patient Information

1 NRIC ☐☐☐☐☐☐ – ☐☐ – ☐☐☐☐

2 Gender

Male (A) (B) Female

3 Ethnicity

Malay (A) (C) Indian

Chinese (B) (D) Others

Section 2 - Inclusion and Exclusion Criteria

Inclusion Criteria

1 Renal hyperparathyroidism — Yes (A) No (B)

2 Single lesion surgically removed
Lesion either adenoma or carcinoma — Yes (A) No (B)

Exclusion Criteria

3 Oestrogens / Raloxifen — Yes (A) No (B)

4 Age < 16 years — Yes (A) No (B)

5 Pregnant — Yes (A) No (B)

Others

6 Vitamin D derivatives — Yes (A) No (B)

Detachable Margin

Detachable Margin

Section 3 - Pre-operative data

1 Patient age

(Use highest level of iPTH, calcium, and ALP where more than one result exists)

2 iPTH (pmol/L) .

3 Corr. Calcium (mmol/L) .

4 Calcium (mmol/L) .

5 Albumin (g/L) .

6 ALP (U/L)

7 Lesion size (cm)

. width . depth . height

OR . cc

8 Bisphosphonate used

Yes No
(A) (B)

[Subject]

Detachable Margin

Section 4 - Post-operative data

1 Date of operation

Day ☐☐ 0 1 2 3 4 5 6 7 8 9 / 0 1 2 3 4 5 6 7 8 9

Month ☐☐ 0 1 2 3 4 5 6 7 8 9 / 0 1 2 3 4 5 6 7 8 9

Year ☐☐ 0 1 2 3 4 5 6 7 8 9 / 0 1 2 3 4 5 6 7 8 9

2 Discharge Date

Day ☐☐ 0 1 2 3 4 5 6 7 8 9 / 0 1 2 3 4 5 6 7 8 9

Month ☐☐ 0 1 2 3 4 5 6 7 8 9 / 0 1 2 3 4 5 6 7 8 9

Year ☐☐ 0 1 2 3 4 5 6 7 8 9 / 0 1 2 3 4 5 6 7 8 9

3 Date Calcium Stable

Day ☐☐ 0 1 2 3 4 5 6 7 8 9 / 0 1 2 3 4 5 6 7 8 9

Month ☐☐ 0 1 2 3 4 5 6 7 8 9 / 0 1 2 3 4 5 6 7 8 9

Year ☐☐ 0 1 2 3 4 5 6 7 8 9 / 0 1 2 3 4 5 6 7 8 9

4 Trough Calcium (mmol/L)
(Lowest documented calcium level in the immediate post-operative period)

☐☐ . ☐☐

[Subject]
