# Supplementary material for: A new method of mark detection for software-based optical mark recognition
Source: PLoS One. 2018 Nov 9;13(11):e0206420. doi: 10.1371/journal.pone.0206420 (PMC6226159; doi:10.1371/journal.pone.0206420)
Supplement: S2 Fig — (PDF) [file pone.0206420.s002.pdf]

Public Survey on Health and Diet

Section 1 - Information

INTRODUCTION What is the purpose of this survey?

This survey is intended to gather information in order to understand the general lifestyle and diet of Malaysian public. The purpose of this is to raise health awareness and to create better education programs. It will also be used to test a new Form Processing Application Software that is designed to help researchers collect data.

PROCEDURE What should I do?

Your participation in this survey is voluntary. If you agree to participate, you are required to complete the following questionnaire. You are encouraged to answer all questions. You can also choose not to answer any questions that make you feel uncomfortable. The information gathered is based on standard questionnaires which have been established by researchers.

RISK AND BENEFIT Are there risks and benefits of participation?

We expect that the risks of participation are negligible.

COMPENSATION Will I receive payment?

While your participation is appreciated, no monetary compensation will be given for completing this survey.

CONFIDENTIALITY

Any information obtained from this survey will only be used for research purposes. Your identity will remain a secret if the study results are published. To ensure confidentiality, you will be given a unique ID as a record in this investigation.

INQUIRY

For further information about this study, please contact the Principal Investigator, Ms PH Tan at +6012-312 5544 or e-mail to *peckhoontan@yahoo.com.sg.*

I have read / been informed and understand the information above and give my consent to participate in this study.

Date: ☐☐ - ☐☐ - ☐☐☐☐

Respondent's Name

Respondent's Signature

Detachable Margin

[Subject]

Detachable Margin

Section 2 - Instructions on Completing the Form

Fill in boxes with capital letters and numbers only. Do not write outside the boxes.

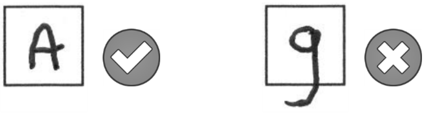

Mark choice tablets clearly using a soft pencil. Do not extend marks into adjacent tablets. Rub out all corrections with a soft eraser.

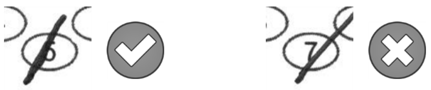

Only marks within a tablet are detected, so avoid circling.

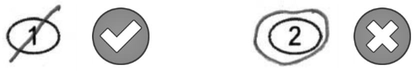

Fill 'box choices' by entering numbers clearly in the boxes and ticking the corresponding tablet.

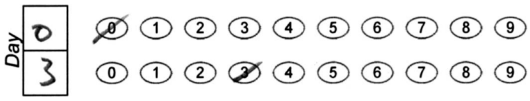

Section 3 - Personal Information

Instructions: The following questions provide information on your personal background. Please tick the single most appropriate answer.

1 Age

Years
(0) (1) (2) (3) (4) (5) (6) (7) (8) (9)
(0) (1) (2) (3) (4) (5) (6) (7) (8) (9)

2 Ethnicity

(A) Malay (B) Chinese (C) Indian (D) E. Malaysia Bumiputra (E) Others

3 Gender

Male (A) Female (B)

4 Marital Status

Never married (A) Married (B) Divorced (C) Widowed (D)

5 **What is your estimated total gross monthly household income?**

RM
(0) (1) (2) (3) (4) (5) (6) (7) (8) (9)
(0) (1) (2) (3) (4) (5) (6) (7) (8) (9)
(0) (1) (2) (3) (4) (5) (6) (7) (8) (9)
(0) (1) (2) (3) (4) (5) (6) (7) (8) (9)
(0) (1) (2) (3) (4) (5) (6) (7) (8) (9)

Detachable Margin

6 What is your estimated total monthly household expenditure?

RM

☐	0	1	2	3	4	5	6	7	8	9
☐	0	1	2	3	4	5	6	7	8	9
☐	0	1	2	3	4	5	6	7	8	9
☐	0	1	2	3	4	5	6	7	8	9
☐	0	1	2	3	4	5	6	7	8	9

7 Years of Formal Education

(1) (2) (3) (4) (5) (6) UPSR (7) (8) (9) LCE/SRP/PMR (10) (11) MCE/SPM

(12) (13) HSC/STPM/Sijil (14) (15) (16) Bachelor (17) (18) Masters (19) (20) (21) PhD

Professional Qualification

Section 4 - Health Information

Instructions: The following questions assess your knowledge of health information. Please tick the single most appropriate answer.

1 Do you consume vitamin supplements of any type?

- (A) Yes (please go directly to Question 2)
- (B) No (please go directly to Question 3)

2 What the most important factor that influences you to consume vitamin supplements?

- (A) Peer pressure.
- (B) Eating supplements is good for health maintenance.
- (C) Doctor’s prescription.
- (D) Addressing a particular health problem.
- (E) Price of vitamin supplements is reasonable compared with the benefits.
- (F) Easy availability of vitamin supplements in the market.

3 What is the biggest reason that influences your decision NOT to consume vitamin supplements?

- (A) Cost is too high for my budget.
- (B) I don’t believe in eating any health supplements. It is better to eat natural food.
- (C) I have no idea how to choose a particular vitamin supplement as there are too many in the market.
- (D) All vitamin supplements are synthetically produced and contain potentially harmful fillers, colouring, lubricants and binders.
- (E) There is no natural solution in the market that satisfies my need.
- (F) Others. Please specify:

Detachable Margin

4 ***Good bowel movement*** *is defined as a minimum of one or more bowel movements per day. Stools quality should be in the shape of banana, golden brown, with adequate bulk and not too smelly. Please describe your typical daily bowel movement.*

- (A) Once a day but the stool quality is not as described above
- (B) More than once a day but the stool quality not as described above
- (C) Once in every two days of more but the stool quality not as described above
- (D) I usually have constipation problem
- (E) Once or more a day with the stool quality similar to the above described
- (F) Others. Please specify:

5 Do you consume probiotics?

- (A) Yes (Please go directly to Question No. 6)
- (B) No (Please go directly to Question No. 7)

6 What are the factors that influence your decision to consume probiotics regularly? (Select all that apply)

- (A) I believe that probiotics will help my digestive system to be more effective and to minimise the risk of related illnesses.
- (B) The cost is reasonable compared to the benefits that I will get in the long term to maintain my health.
- (C) The various marketing and advertisements that I am exposed to reinforces the importance of probiotics
- (D) Probiotics are easily available in the market.
- (E) Others. Please specify:

7 What are the factors that influence your choice NOT to consume probiotics? (Select all that apply)

- (A) I don't know what probiotics are.
- (B) I do not believe in consuming probiotics. I believe my body produces sufficient good and friendly bacteria.
- (C) I do not have the time to source and find the right/suitable probiotics.
- (D) I am confused with too many health supplements in the market. Hence, I have decided not to take any of them.
- (E) The prices of probiotics are too high for my budget.
- (F) My hectic lifestyle or work makes me tend to forget to consume any supplementary health products.
- (G) Others. Please specify:

8 *Fibre is defined as roughage usually found in fruits and vegetables that cannot be digested fully by our stomach but vital to our health. Do you consume fibre everyday?*

- (A) Yes (Please go directly to Question No. 9)
- (B) No (Please go directly to Question No. 10)

Detachable Margin

9 How much fibre do you consume per day?

- (A) Minimum an apple or equivalent fruit/vegetable of the same size/portion a day
- (B) Option (a) above plus some green vegetables (5-6 strands or long leaves or equivalent) a day.
- (C) A medium size plate of green vegetables or a bowl of salad everyday – estimate of 4 apples worth.
- (D) At least 1 kg of green vegetables or equivalent fruit a day.
- (E) Less than option (a).
- (F) Others. Please specify:

10 What are the factors that influence your decision NOT to consume fibre? (Select all that apply)

- (A) I don’t understand the benefits of fibre to my body.
- (B) I am too busy to take any additional health related products whether natural or otherwise.
- (C) Price of fibre is too expensive for my budget.
- (D) I don’t like the taste of fibre (fruits and vegetables, etc) in my mouth.
- (E) I can never eat enough fibre, so it's best not to eat any at all.
- (F) Others. Please specify:

11 *According to the British Nutrition Foundation, an adult of 18 years or more needs 30g daily intake of fibre. This is equivalent to 15 apples a day. Note: An apple with its skin is approximately 2.4g fibre each.* Are you aware of this?

- (A) Yes
- (B) No

12 How many times a year (on average) do you fall sick? *Sickness is defined as illness that resulted in you not being able to carry out your normal routine (employment or otherwise).*

- (A) At least once a month
- (B) 1-3 times a quarter (3 months)
- (C) 1-3 times half yearly
- (D) 1-3 times annually
- (E) Others. Please specify:

13 What steps do you take if you catch a common cough, flu or fever?

- (A) Self medication of western medication. Examples: Take Panadol, cough or flu medication based on previous history of certified doctor’s prescription.
- (B) Self medication via traditional medicine or herbs.
- (C) Seek strict medical attention from the local certified western doctor and take the medication prescribed.
- (D) Take plenty of rest and hope that the body will heal by itself.
- (E) Combination of (a) and (b).
- (F) Combination of (a) an (b). If it does not work, I will go for option (c).

14 What are the most important factors that would make you seek a western doctor for treatment when you fall sick? (Select all that apply. Leave blank if not applicable)

- (A) Traditional herbs and drugs are too unreliable and not proven.
- (B) Western trained doctors are the best possible options for mankind.
- (C) Medical treatment is covered by my company's insurance provider, so I am best served being treated by the western doctors.
- (D) I have had a bad experience using traditional/alternative treatment.
- (E) Traditional herbs and medication need to be free from toxin, certified by third parties before I am open to using it.
- (F) I believe in alternative/traditional treatment, but I have no idea where to seek such reliable services.
- (G) Others. Please specify:

Section 5 - Physical Activity (Short IPAQ)

Instructions: The following questions provide information on your level of physical activity. Please fill in the relevant boxes with numbers indicating the amount of time spent on a particular type of activity.

1 During the last **7 days,** on how many days did you do **vigorous** physical activities like heavy lifting, digging, aerobics, or fast bicycling?

Days per week (if none, skip to Q3) ☐

2 How much time did you usually spend doing **vigorous** physical activities on one of those days?

Hours per day ☐ Minutes per day ☐ Don't know / Not sure ◯ Tick if applicable

3 During the last **7 days,** on how many days did you do **moderate** physical activities like carrying light loads, bicycling at a regular pace, or doubles tennis? Do not include walking.

Days per week (if none, skip to Q5) ☐

4 How much time did you usually spend doing **moderate** physical activities on one of those days?

Hours per day ☐ Minutes per day ☐ Don't know / Not sure ◯ Tick if applicable

5 During the last **7 days,** on how many days did you **walk** for at least 10 minutes at a time?

Days per week (if none, skip to Q7) ☐

6 How much time did you usually spend **walking** on one of those days?

Hours per day ☐ Minutes per day ☐ Don't know / Not sure ◯ Tick if applicable

7 During the last **7 days,** how much time did you spend **sitting** on a week day?

Hours per day ☐ Minutes per day ☐ Don't know / Not sure ◯ Tick if applicable

Detachable Margin

Detachable Margin

Section 6 - Diet (Dietary Diversity)

Instructions: The following questions provide information on the quality of your diet. Please describe the foods (meals and snacks) that you ate yesterday during the day and night, whether at home or outside the home. Fill in the box with what you ate for that meal, and tick the tablets below to indicate what the meal contains.

Example Chicken rice

(A) Grain (B) Vegetables (C) Dairy (D) Fruit (E) Meat

Meat: fish, chicken, beef, egg

Dairy: milk and milk products

Grain: bread, rice, cereal, noodles

1 Breakfast

(A) Grain (B) Vegetables (C) Dairy (D) Fruit (E) Meat

2 Lunch

(A) Grain (B) Vegetables (C) Dairy (D) Fruit (E) Meat

3 Dinner

(A) Grain (B) Vegetables (C) Dairy (D) Fruit (E) Meat

4 Supper

(A) Grain (B) Vegetables (C) Dairy (D) Fruit (E) Meat

5 Snacks

(A) Grain (B) Vegetables (C) Dairy (D) Fruit (E) Meat
