# Supplementary material for: A new method of mark detection for software-based optical mark recognition
Source: PLoS One. 2018 Nov 9;13(11):e0206420. doi: 10.1371/journal.pone.0206420 (PMC6226159; doi:10.1371/journal.pone.0206420)
Supplement: S3 Fig — (PDF) [file pone.0206420.s003.pdf]

IEEE Access
Multidisciplinary ⋮ Rapid Review ⋮ Open Access Journal

Received May 22, 2017, accepted June 20, 2017, date of publication June 23, 2017, date of current version July 17, 2017.

Digital Object Identifier 10.1109/ACCESS.2017.2719284

A Software Application for Survey Form Design and Processing for Scientific Use

SENG CHEONG LOKE[1,2], KHAIRUL AZHAR KASMIRAN[3], AND SHARIFAH AZIZAH HARON[2,4]
[1]Faculty of Medicine and Health Sciences, Universiti Putra Malaysia, Serdang 43400, Malaysia
[2]National Gerontology Research Institute of Malaysia, Serdang 43400, Malaysia
[3]Faculty of Computer Science and Information Technology, University Putra Malaysia, Serdang 43400, Malaysia
[4]Faculty of Human Ecology, University Putra Malaysia, Serdang 43400, Malaysia

Corresponding author: Seng Cheong Loke (lokesengcheong@gmail.com)

This work was supported by the Swan Foundation, Malaysia.

ABSTRACT A form processing application (FPA) automates digitization of information contained in forms. Smaller research groups do not use FPAs as they cannot justify operation of an in-house commercial system. This paper describes the design and testing of a new FPA that is targeted toward the needs of this group, and is released as free open-source software. The new FPA covers form design, printing, scanning, and digitization. It has a flexible plug-in architecture and double-keying is used to reduce transcription error. A common content module (CCM) implements the form design based on the format-independent hierarchical content. The scan module has basic handwriting recognition and can process the input fields used by the CCM. The FPA was field-tested using data from a clinical study to compare the error rate with manual processing. A similar comparison was also made between interviewer and self-administered survey forms. The first comparison shows that the FPA with double-keying had no errors while manual transcription had three errors (0.06%) out of 4952 input fields (p=0.083). The second comparison shows that the FPA with double-keying had no errors for the interviewer-administered form (0/3681 fields, 0%), while the self-administered form had three errors (3/6096 fields, 0.05%) (p=0.178). When double-keying was not used, the error rate for tablet-type fields was not significantly different (p=0.120) between the interviewer (2/3400 fields, 0.06%) and self-administered forms (19/6000 fields, 0.32%). There was, however, a highly significant difference (p<0.001) for handwriting-type fields between the interviewer (11/881 fields, 1.25%) and self-administered forms (75/1896 fields, 3.96%).

INDEX TERMS Document handling, handwriting recognition, image matching, image processing, image registration, object detection, optical character recognition software, pattern recognition, printers, software algorithms.

ABBREVIATIONS AND ACRONYMS

CCM	Common Content Module
DBN	Deep Belief Networks
FPA	Forms Processing Application
ICR	Intelligent Character Recognition
kNN	k-Nearest Neighbors algorithm
MyAgeing	National Gerontology Research Institute of Malaysia
OMR	Optical Mark Recognition
SVM	Support Vector Machines
WIA	Windows Image Acquisition

I. INTRODUCTION

Community surveys are the bread and butter of social science. The usual workflow involves designing survey forms, dispatching enumerators to interview people and record their responses, digitizing the information into a database or spreadsheet, data cleaning to correct errors, and finally analyzing the output.

Clinical studies also make use of survey forms to record information from patient case notes prior to data entry. This is normally done by the clinicians or staff members, which is both time-consuming and prone to error, given the varying degrees of research experience and computer expertise they typically have [1].

The entire process of manually transcribing data from survey forms is very costly and manpower intensive, especially for large community surveys with sample sizes into the thousands. This process is also prone to typographical errors which necessitate stringent cleaning to preserve data integrity. The use of computers, scanners, and forms processing software can automate much of

2169-3536 © 2017 IEEE. Translations and content mining are permitted for academic research only.
Personal use is also permitted, but republication/redistribution requires IEEE permission.
See http://www.ieee.org/publications_standards/publications/rights/index.html for more information.

this work, thus saving costs and reducing manpower requirements.

An experienced data entry operator can process about 30-50 hour-long survey forms per workday. Modern document scanners can process 2-3 such survey forms per minute for mid-range desktop models, and 5-15 forms per minute for commercial production versions [2].

A. FORMS PROCESSING APPLICATION

A Forms Processing Application (FPA) handles the entire process of scanning survey forms, digitizing the information, and converting this into a spreadsheet or storing it in a backend database. The scanned images cannot be directly used and need to be first digitized through a variety of recognition methods. Simple checkboxes and bubble marks can be read with Optical Mark Recognition (OMR) technology. Printed characters can be read using Optical Character Recognition, while handwriting can be read using an Intelligent Character Recognition (ICR) system [1].

Currently, most survey research and clinical studies carried out by smaller departments and groups do not make use of FPAs due to the costs involved [3]–[6]. The National Gerontology Research Institute of Malaysia (MyAgeing), a multidisciplinary institution that covers social science, medical, and engineering approaches to ageing research, relies heavily on survey forms for its research needs. It indirectly makes use of FPAs for its large community surveys by outsourcing the scanning and digitization process to commercial providers [7]–[9]. This may however compromise data security, especially when sensitive personal and health-related information is collected.

An in-house FPA system would ideally allow MyAgeing to take control over these processes, but the setup costs for the software, associated hardware, and initial training are substantial. There will also be additional costs such as for manual data entry fallback, database and form design, annual support contracts and warranties, and further training for new staff [6].

The current industry leader TeleForm by Hewlett-Packard has been employed in a number of large epidemiological surveys and clinical studies, and found to be sufficiently accurate for scientific use [10]–[12]. It has however been documented to have significant technical issues such as software bugs and incompatibilities with other programs especially with version upgrades. Practical issues such as the need for pre-scan checking of forms as well as un-stapling questionnaires prior to scanning and re-stapling them post-scanning are extremely time consuming and tedious, and may offset most of the gains from automation [6]. Mechanical issues such as scanner paper jams and missing registration marks further erode productivity due to the need for re-scanning or manual data entry [6], [11]. The software is also complex and requires special training for personnel involved with data entry and scanning [11].

In view of the above, the authors have designed a new FPA to cater for the research needs of MyAgeing. The FPA will also be released as open-source software under the GNU General Public License version 3, which will allow other researchers to use and adapt the software at no cost. This article describes the functions and characteristics of the new FPA, as well as the results of field-testing with forms from actual clinical and community-based studies.

B. LITERATURE SEARCH

A comprehensive literature search yielded 38 articles and 6 book sections which were relevant to FPAs, out of a total of 499 articles and 79 book sections initially screened. Approximately two thirds of these articles were older than 15 years, and only one out of eight were recent articles published less than 5 years ago. The age distribution of the articles suggests that this field of research is mature and relatively little has been done in recent years. Most of the recent articles focused on research experience with TeleForm, and surfaced numerous issues as detailed above.

II. DESIGN OVERVIEW

The main priority for the new FPA was to keep the interface as simple and user-friendly as possible, while maintaining the core functions of form design, scanning, and digitization. Another priority was to implement a flexible plug-in architecture so that new capabilities and upgrades can be easily incorporated in the future without disrupting existing functionality. If this is executed correctly, version upgrades will be easier to debug and less prone to the technical and compatibility issues that plague other FPAs.

All modules were to have a common design motif with a shared messaging area onscreen to allow them to communicate with the user. As the FPA was targeted at small scale users, no security processes or audit markers were planned as this would adversely impact on ease of use and increase the complexity of the software. The software was designed for the Microsoft Windows platform as most of the personal computers used by smaller departments and individuals are of this type.

A. PLUG-IN ARCHITECTURE

The architecture is designed around a main program with upgradable plug-ins that provide most of the functionality of the software. The main program is a normal executable file, while the plug-ins are dynamic-link libraries that reside in a subdirectory of the executable file.

Communication between the main program and plug-ins is limited to a lightweight interface which controls initial activation of the plug-ins, a common variable pool, shared user messaging, and persistent storage of configuration parameters. Upgrades to functionality can be performed by deleting the old plug-in subdirectory and copying in the new one. The main program can also be updated whenever the interface motif is redesigned. However, it will still have to honor the established communication protocol with the plug-ins.

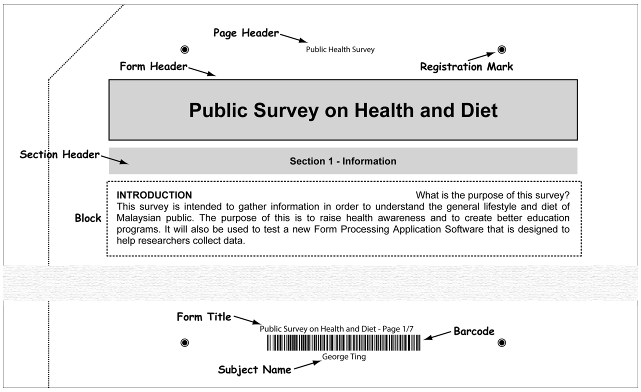

FIGURE 1. Extract of the first page of a survey form designed in the CCM showing typical structural elements.

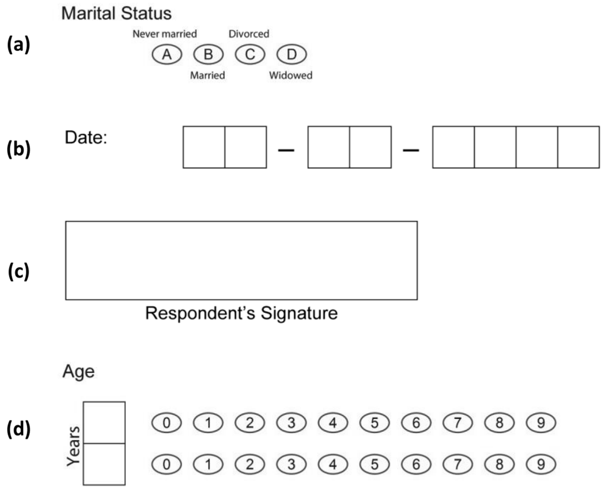

FIGURE 2. Examples of predefined field types. The field types shown are: (a) choice field, (b) handwriting field, (c) free entry field, and (d) box choice field.

B. COMMON CONTENT MODULE

The Common Content Module (CCM) is a designer that specifies the format-independent hierarchical content of a form. The smallest unit in the hierarchy is the field which defines content of a single type. One or more fields make up a block, which typically represents a single form item. A block can take up either a half or full page width, and cannot be broken up across page boundaries. Sections are made up of multiple blocks and usually correspond to stand-alone content such as demographics and epidemiological instruments. These sections can be designed once and subsequently reused to rapidly prototype forms.

A form has headers and footers that appears on every page, and a form header that is only shown at the beginning of a form. The form header consists of one or more stacked blocks, typically with text and image content. The page header is a simple text label, while the footer contains a barcode with information about the study name, page number, and subject name (Fig. 1). The barcode type used is the GS1-128 Code Set B symbology as this is a high density alphanumeric standard format that can be recognized by most software barcode readers [13].

There are several predefined field types which can be classified as either for input or non-input. Input fields are filled by the respondent and correspond to data items once the form contents have been digitized. They can be multiple choice bubbles, handwriting boxes, free input fields, or box choice fields (Fig. 2). Non-input fields can contain either text or an image, and typically provide instructions for completing the item. Formatting fields affect how blocks and sections are displayed, and include page breaks, dividers, item groups, borders, backgrounds, and numbering.

Sub-section and multiple choice fields provide templates which are filled by content imported from an external Microsoft Excel spreadsheet in predefined formats. This allows large repeating units such as a list of multiple choice questions to be rapidly designed with a minimum of effort.

Forms are designed with a detachable margin of 1.5 cm and a diagonal 2.5 cm cut-out which allows the pages to be stapled either on the on the upper left corner or left border (Fig. 1). Before scanning, the margin can be removed from a stack of forms using a paper guillotine without having to touch the staples. There is a lower and upper scan margin of 1.5 cm to accommodate scanner misfeeds.

C. CCM INTERFACE

The CCM interface is split into three vertical panels, with the left panel showing a hierarchical content tree, the middle panel showing a preview of the item selected from the content tree, and the right panel showing the building blocks available for the selected item (Fig. 3). The building blocks are dragged from the right panel to the left panel to construct a form, and the contents can be customized and previewed from the middle panel. Blocks can also be copied or moved from within the left panel to replicate existing sections.

The CCM module is optimized for mouse and keyboard input, although users with touch screens can drag and drop blocks. Icons and other user interface elements are designed to be relatively large and distinctive, with integrated tooltips and a help function to guide new users. The help function is activated by dragging a user interface element onto the help button at the bottom right of the screen.

The preview image is generated live from the content, and any changes such as typing in text or reordering blocks is reflected immediately. These design priorities are intended to ease the learning curve for the intended target userbase, which will not have the resources to spend on training or employ dedicated data entry personnel.

Once a study form has been designed, a list of subjects can be imported from an Excel spreadsheet. Based on this list, PDF copies of the study form are generated with individualized bar codes. As the form layout is automatically determined by the FPA, any changes during a study can be

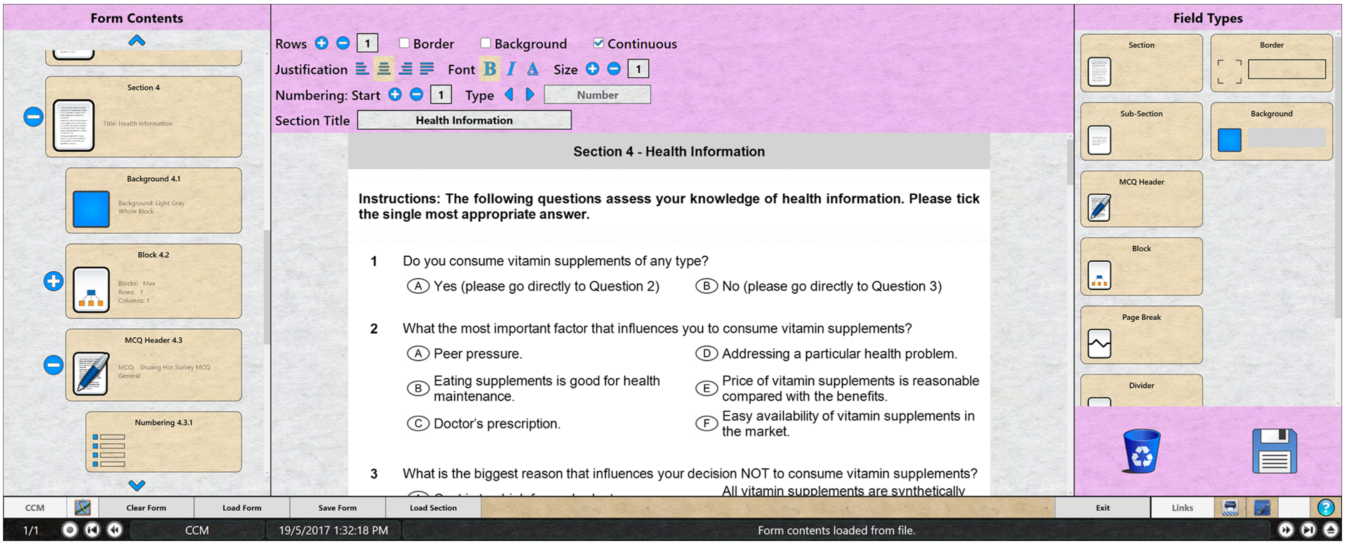

FIGURE 3. The Common Content Module showing a section preview.

implemented without having to redesign the entire form. The use of formatting fields allows some degree of control over the automatic form layout process. This gives the benefit of having formatting templates without the complexity of implementing and using them [14].

D. SCAN MODULE

For most FPAs, image acquisition is through a dedicated scan function that controls a document scanner. Software programs communicate with scanner drivers through an abstraction layer, of which there are three main standards: 1) TWAIN that is supported by older scanners, 2) Image and Scanner Interface Specification that is used mainly by document scanners, and 3) Windows Image Acquisition (WIA) which is supported by newer consumer scanners [15].

The scan module offers direct scanning with TWAIN and WIA support, which covers most scanners being used by smaller departments and individual researchers. The module also features direct importing of image files into the FPA. This allows scanners without TWAIN, WIA support, or outdated drivers to still act as sources for the FPA through their own dedicated image capture applications. During the scan process, backup image files are also generated so that if there is any interruption, these images can be imported into the module without having to rescan.

E. SCAN MODULE INTERFACE

The scan module interface is split into a left vertical panel and two right horizontal panels. The left panel shows either a summary table of the detected marks from each input field or a PDF preview from which a field can be directly selected. The upper right panel shows the scanned image from the selected input field while the lower right panel shows the corresponding detected marks (Fig. 4). The detected marks can be changed by the operator either from the summary table or the lower right panel.

On the left margin are icons which control the scan process and filter the displayed items on the summary table. The filters can limit the displayed items to those either with or without data, and show critical fields for double keying. Fields for double keying can be changed between automatically detected marks, operator entered marks, and the final marks which have been reconciled by the supervisor.

The top margin contains navigation icons which allow movement to subject records either by number, name, or presence of input fields which need user verification. The dropdown menu allows fast navigation as subjects requiring verification are highlighted in red.

III. MARK RECOGNITION

Scanned images are de-skewed with the assistance of preprinted registration marks (Fig. 1). These registration marks are of a new design with high detection strength and accuracy, and are resistant to image rotation, noise, and scan artefacts [16].

Current methods of mark detection for bubble input fields require that either the bubbles be completely darkened, have no internal labels, or be clearly filled with a black pen or soft pencil. This FPA uses a new detection algorithm that dispenses with these requirements and yet maintains a high level of accuracy [17].

Handwriting fields are first cut out individually from the scanned image (a). A narrow border mask is applied (b) to remove the surrounding box and give the raw character image (c). White space surrounding the character is removed (d) and the image resized and contrast adjusted to show the standard character image (e). The handwriting from different individuals tends to skew at a different angle (f). Correcting the skew is done by vertical segmentation of the image and determining the center of gravity for each segment. A fit line is drawn through the segment centers and this gives the angle of skew (g)

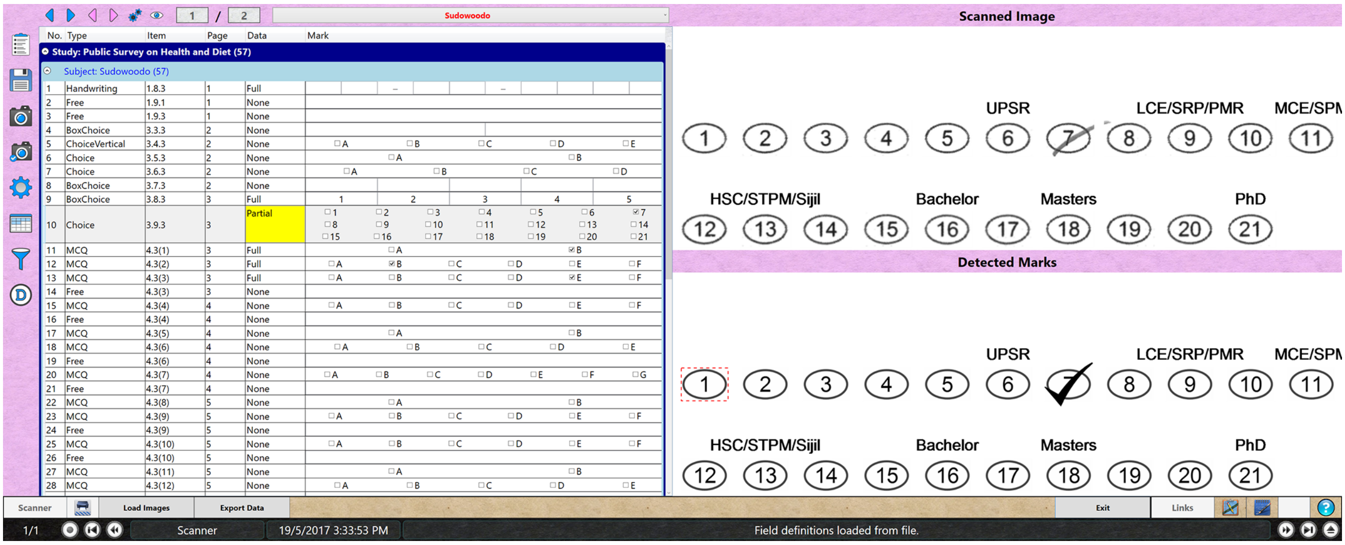

FIGURE 4. Scan Module showing a table of input fields and marks from the currently selected field.

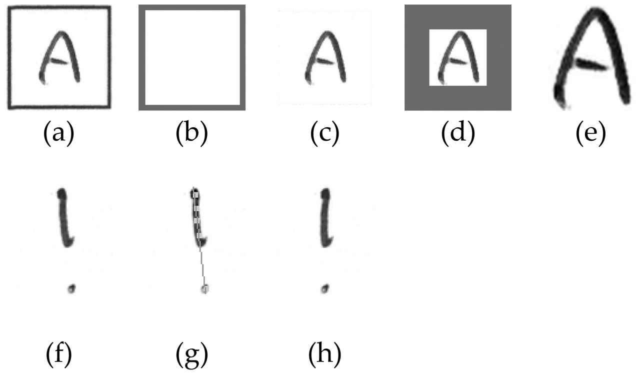

FIGURE 5. Initial processing steps for handwriting fields.

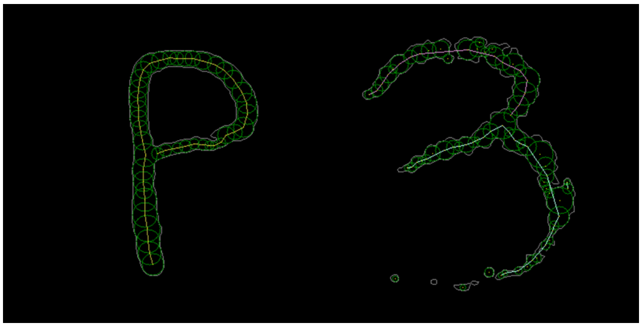

FIGURE 6. The segmentation routine used to extract strokes from character images.

which is then corrected to give the de-skewed character image (h) (Fig. 5).

These character images are then converted into vectors arrays using a set of transformation routines. Each transformation routine extracts a set of vectors that describe the character in a particular way. The first routine derives the intensities of each pixel within the character image as a single vector. The second routine extracts the histogram of gradients within the image as vectors.

The last routine uses segmentation to extract strokes as vectors. This is done by converting the character image into contours, and using circles to determine the centerline of each contour. Individual strokes are isolated from the centerlines by identifying intersections where more than two circles come into contact (Fig. 6).

Limited ICR capability is provided for handwriting input fields. As the error rate for handwriting recognition is relatively high, multiple routines are used for detection and a voting system implemented to select the output. The three recognition routines used are the k-Nearest Neighbors algorithm (kNN), Support Vector Machines (SVM), and Deep Belief Networks (DBN). These routines are different implementations of unsupervised (kNN) and supervised (SVM, DBN) machine learning which have been successfully used in character recognition.

Image recognition routines are pre-trained using sample handwriting templates that are collected from volunteers. Standard character images are first extracted from the templates, and screened to remove those which fail a quality check. The quality algorithm flags characters that are written out of the box, or which have extensions from neighboring boxes. The remaining characters are then converted into vectors arrays in the same manner as scanned images.

When trained and tested on a dataset containing handwriting samples collected from volunteers, alphanumeric handwriting fields were found to have low-moderate accuracy of about 77%, while purely numeric fields have an accuracy of about 99% (Table 1).

The accuracy of alphanumeric fields can be increased by post-processing of the results. This was done by segmenting each field into words, and if a word began and ended with either a number or alphabet, it was assumed

TABLE 1. Percentage accuracy for handwriting recognition based on different vector transformation-recognition engine combinations.

Recognition Engine	Vector Transformation Routine		
%Accuracy	Intensity	HoG	Stroke
Numeric			
KNN	**100**	**97.6**	88.9
SVM	**94.8**	**97.0**	**95.9**
Deep	N/A	**96.8**	**95.8**
Alphanumeric and Punctuation			
KNN	**68.0**	**70.2**	52.2
SVM	**68.2**	**74.1**	**70.1**
Deep	N/A	**69.2**	**65.9**

Note: Intensity=raw pixel values, HoG=histogram of gradients, Stroke=stroke segmentation. KNN=k-nearest neighbors algorithm, SVM=support vector machines algorithm, Deep=deep belief networks algorithm. The percentage accuracy was calculated as the count of correctly identified characters divided by the total image count.
Numeric: Training was done using a dataset with 5745 images, and tested on a separate dataset with 830 images. The pooled accuracy from the highlighted combinations was 99.2%.
Alphanumeric-punctuation: Training was done using a dataset with 44746 images, and tested on a separate dataset with 6589 images. The pooled accuracy from the highlighted combinations was 77.0%.

that the other characters were also numbers or alphabets respectively. While not always accurate, this routine helps to differentiate between commonly confused number-alphabet pairs which account for about 70% of the total alphanumeric error. Dictionary-based post-processing can potentially further improve on the results, but this was not implemented in the current version of the FPA [18].

Errors in data entry are common in large studies with a rate of about 0.1-1.0% depending on the type of field. Error rates can be lowered by entering the data a second time and validating against the original entry. This process of double keying can lower the error rate by up to five times and is the method of choice when data accuracy is paramount [19].

IV. FIELD TESTING

The target user base for the new FPA are individual researchers and small departments which currently rely on manual forms processing for their clinical studies and community surveys. As the FPA is to be released as free software, its cost is comparable to that of existing manual processes, being derived mostly from manpower and training. Aside from costs, another important aspect which can be examined is the error rate with the new FPA compared with existing manual processing.

A. METHODOLOGY

During field testing, the comparison of error rate was done by redesigning the study form for a previous clinical study using the CCM while retaining its contents. Data from the actual study was then used to fill the input fields by hand. The forms were inspected and the results manually transcribed into a spreadsheet. The new FPA was used to digitize the same forms into a separate spreadsheet. The results from both spreadsheets were compared and any discrepancies resolved by examining the original forms. The error rates for manual and automatic processing were then calculated on a field-by-field basis.

Community surveys can either be interviewer-administered or self-administered, and the performance of the new FPA needs to be examined in both situations. A direct comparison was done by designing a new form using sections from previous studies [7]–[9]. These sections were selected to be compatible with both interviewer and self-administration. Subsequent data processing followed the same algorithm outlined above for the clinical study form.

The clinical study used was the "Renal Hyperparathyroidism Study" conducted at Hospital Sultan Ismail from 2011-2015 with data from 85 subjects [20]. The community survey was the "Health Perceptions Among the Malaysian Public" survey. The self-administered survey was run on 75 subjects recruited from a public location, while the interviewer-administered survey was run on 50 subjects recruited from a single housing area. A convenience sample was used rather than random sampling, as the actual information was not the item of interest, only the error rate for data entry.

The self-administered survey was distributed by two untrained volunteers with no instructions given to respondents other than that printed on the forms. The interviewer-administered survey was run by a single trained enumerator who filled the forms in her own handwriting. This community survey was granted exemption from ethics review (UPM/TNCPI/RMC/1.4.18.1(JKEUPM)/004) while the clinical study was granted full ethics approval (KKM/NIHSEC/P15-617)(NMRR-15-554-24819 (IIR)).

B. STATISTICAL TREATMENT AND SAMPLE SIZE DETERMINATION

The primary analysis was powered to show non-inferiority of the FPA compared to manual processing in terms of error rate. The secondary analysis was powered to show the difference in error rate for the FPA when comparing the self-administered and interviewer-administered survey forms.

The statistical test used for the primary analysis was a non-parametric paired T-test (Wilcoxon signed-rank test). As only non-inferiority was looked for, the test was one-tailed. The

TABLE 2. Comparison of error rates comparing the new FPA and manual data transcription for the clinical study according to field type.

	Manual		FPA		P-Value
	N	%	N	%	
**Handwriting**					
Errors	2	0.075	0		0.157
Field Count	2657	±0.019	2657		
**Choice Horizontal**					
Errors	0		0		N/A
Field Count	595		595		
**Choice Vertical**					
Errors	0		0		N/A
Field Count	170		170		
**Box Choice**					
Errors	1	0.065	0		0.317
Field Count	1530	±0.026	1530		
**OVERALL**					
Errors	3	0.061	0		0.083
Field Count	4952	±0.014	4952		
**COMBINED BOXES**					
Detection Errors			35	0.836±0.015	
User			1	0.024±0.015	
Software			34	0.812±0.015	
Field Count			4187		
**COMBINED TABLETS**					
Detection Errors			0		
User			0		
Artefacts			0		
Software			0		
Field Count			2295		

Note: The p-value was derived using the Wilcoxon signed-rank test where there was a difference in the error rate. For purposes of clarity, the percentage error rate is given only for non-zero rates with the associated error bounds. The overall error rate for the FPA is taken from the final reconciled results after double-keying. The rates for combined boxes and tablets are the raw error rates before double-keying.

test used a preset 5% type I error rate (alpha-level 0.05), and a 5% type II error rate (95% power), looking for a 1% difference with a standard deviation of 2.5%, which worked out to a Cohen's d value of 0.40. Power analysis calculated using G* Power 3.1 based on the above gave a minimum sample size of 73 forms for the clinical study [21], [22].

The statistical test used for the secondary analysis was a two-tailed non-parametric unpaired T-test (Wilcoxon-Mann-Whitney test). This time, a 10% type II error rate (90% power), looking for a 1.5% difference, standard deviations of 2.5% and 2.0% in each group respectively, and a 1:1.5 allocation ratio, worked out to a Cohen's d value of 0.66.

For the interviewer-administered study form, we expected a lower variability as fewer people were involved, so the set standard deviation was reduced to 2%. Power analysis gave a minimum sample size of 65 forms for the self-administered survey and 43 forms for the interviewer-administered survey.

C. RESULTS

Based on results from the clinical study, the error rate for the new FPA (0/4952 fields, 0%) after double-keying was not higher ($p=0.083$) compared with manual data transcription (3/4952 fields, 0.06%) (Table 2). The error rate for the new FPA was zero across all field types, thus showing the effectiveness of the double keying process. Manual transcription also performed well, with an overall error rate less than 0.1%, which is acceptable for critical data fields [23].

Without double-keying, the detection accuracy for tablet-type fields was still very high, with no errors in the 2300 input fields processed. On the other hand, the accuracy for handwriting-type fields (35/4187 fields, error 0.84%, accuracy 99.2%) was consistent with results obtained from the test dataset, which was 99.2% (Table 1, Table 2).

The FPA with double-keying had no transcription errors for the interviewer-administered form (0/3681 fields, 0%), while the self-administered form had three errors (3/6096 fields, 0.05%), which was not significantly different ($p=0.178$).

When double-keying was not used, the error rate for tablet-type fields was not significantly different ($p=0.120$) between the interviewer (2/3400 fields, 0.06%) and self-administered (19/6000 fields, 0.32%) survey forms. The error rate for software detection was less than 0.1%, but there were several

TABLE 3. Comparison of error rates between the interviewer-administered and self-administered survey forms when using the new FPA.

	Interviewer		Self		P-Value
	N	%	N	%	
**Handwriting**					
Detection Errors	2	0.712±0.060	33	3.313±0.032	0.018
Verification Errors	0		0		
Field Count	281		996		
**Choice Horizontal**					
Detection Errors	2	0.571±0.053	13	2.476±0.044	0.034
Verification Errors	0		0		
Field Count	350		525		
**Choice Vertical**					
Detection Errors	0		1	0.051±0.023	0.414
Verification Errors	0		0		
Field Count	1300		1950		
**MCQ**					
Detection Errors	0		5	0.190±0.020	0.068
Verification Errors	0		3	0.114±0.020	0.157
Field Count	1750		2625		
**DOUBLE-KEYED FIELDS**					
Verification Errors	0		3	0.049±0.013	0.178
Field Count	3681		6096		
**Box Choice**					
Choice Detection Errors	0		0		
Box Detection Errors	9	1.500±0.041	42	4.669±0.034	0.001
Field Count	600		900		
**COMBINED BOXES**					
Detection Errors	11	1.249±0.034	75	3.956±0.023	<0.001
User	2	0.227±0.034	35	1.846±0.023	0.001
Software	9	1.022±0.034	40	2.110±0.023	0.043
Field Count	881		1896		
**COMBINED TABLETS**					
Detection Errors	2	0.059±0.017	19	0.317±0.013	0.120
User	0		10	0.167±0.013	0.075
Artefacts	2	0.059±0.017	7	0.117±0.013	0.900
Software	0		2	0.033±0.013	0.427
Field Count	3400		6000		

Note: The p-value was derived using the Wilcoxon-Mann-Whitney test where there was a difference in the error rate. For purposes of clarity, the percentage error rate is given only for non-zero rates with the associated error bounds. For the interviewer-administered survey, 67 tablets had detectable artifacts, while the self-administered survey had 88 tablets with artifacts. All input field types were double-keyed with the exception of box choice fields where the tablet and handwriting components were used instead.

errors due to print artifacts caused by "salt and pepper" type deposits. The algorithm for removal of this kind of noise was effective with a 94% success rate (146 out of 155 artifacts filtered out) (Table 3) [17].

The error rate for handwriting-type fields was significantly different (p=<0.001) between the interviewer (11/881 fields, 1.25%) and self-administered (75/1896 fields, 3.96%) survey forms. Software detection accuracy for handwriting-type fields in the interviewer-administered survey was consistent with the test data (99.0% vs 99.2%), but accuracy for the self-administered survey was lower (97.9% vs 99.2%) (Table 1). This is likely because the trained enumerator who ran the interviewer-administered survey took care to write neatly, compared to respondents for the self-administered survey who were often in a rush to complete the forms. For the same reason, the proportion of user-entry errors was much higher for the self-administered survey (handwriting: 1.8% vs 0.2%, p=0.001; tablet: 0.2% vs 0.0%, p=0.075) (Table 3).

There were a small number of verification errors (3/4375 tablets, 0.07%) for the MCQ fields, which was otherwise not present in the other fields or the clinical study. Feedback from the data entry staff member and the

enumerator was that the MCQ answer stems were placed too close to each other and were hard to differentiate.

V. DISCUSSION

The error rate for tablet-type fields is low enough that double-keying is not needed provided the forms are printed properly. For this study, it was noted that printer hardware issues caused several of the community survey forms to be contaminated with wide bands of "salt and pepper" type deposits. A decision was made to deliberately use these mis-printed forms so that the efficacy of the artifact-removal algorithm could be field-tested.

Hence, for clinical study forms and interviewer-administered forms where the user error rate is low, double keying is not required as the overall detection error rate is less than 0.1%. On the other hand, enough user-entry errors were present on self-administered forms such that the overall error rate fell between 0.1-1.0%. This means that double-keying will be required for critical fields, but can be omitted for non-critical fields.

The error rate for handwriting-type fields is high enough (greater than 1%) that double-keying is recommended in all cases. Although the tested error rate where the data entry person wrote neatly was only 0.8%, in routine use this is expected to vary widely.

The quoted error rate for handwriting recognition in the industry-standard TeleForm is 0.4% after optimization with data validation rules and error-checking scripts, which is half that of this FPA (0.8%). In practice, user entry errors will push the overall error rate to more than 1%, so double-keying will still be required when using TeleForm (Table 3).

The combined software error rate for tablet mark detection across all three forms was 0.017 ± 0.009 (2 errors out of 11695 tablets), which compares favorably with the error rate of 0.02% for the OMR component of TeleForm [10]. Tablet mark detection is more difficult in this FPA where tablets can contain an internal character label, compared with TeleForm where all labels are external to the tablets [12].

The page alignment routine used in the FPA is highly robust with pixel-perfect alignment in each of the 1130 pages processed across the three forms. This is much better than the 2% misalignment rate quoted for TeleForm [6], [16].

MCQ questions should be designed such that adjacent answer stems are either spaced out properly, or distinguished using alternate normal and italic fonts. This will facilitate both data entry from interviewers and respondents, as well as data verification.

Box choice questions were originally intended to automatically double-key critical numeric fields. However, the box (handwriting) component was found to have a high error rate (1.5-4.7%) (Table 3), while the choice (tablet) component was error-free. The error rates for the box component were 40-100% higher than the corresponding error rates for handwriting fields (0.7-3.3%), and this could be because respondents were less careful when two different modes of data entry were used. From this it would make sense to discard the box component and instead rely on manual double-keying of the choice component where necessary.

A. STUDY WEAKNESSES

The first study weakness is that a head to head comparison with the FPA industry leader TeleForm should have been done. However, funding was not available to purchase a fully functional version. Similarly, a comparison with commercial handwriting recognition applications could also have been performed if it were not for the high cost of such software.

This weakness is however mitigated by the fact that the targeted user base is different from that of high-end applications such as TeleForm. The key purpose of this research is to design and test an FPA for a group of researchers who would otherwise not have been able to use TeleForm due to cost and other considerations.

The second weakness is that both the data entry and double keying functions were carried out by the study investigator instead of being done by two separate people. This was however partially mitigated by the user interface design of the scan module which blinds the data entry person from seeing the detected mark.

The final weakness is that there is no documented end user feedback on the FPA. However, the usefulness of such feedback in this situation is limited given that the investigator played the role of the sole data entry operator, and only a single enumerator was employed. By releasing the software publicly, it is hoped that this feedback would be obtained once enough people have started to use the software.

B. FUTURE DEVELOPMENT

Based on the findings from this study and the observed shortcomings during field testing, a second version of the software was developed. This has been made available on GitHub together with the source code (https://github.com/scloke/Survey2). A template for development of new modules has also been included. If there is demand for it, a handwriting training module will be released which allows advanced users to train the recognition routines using their own samples. This can also be used for non-Latin character sets such as Thai or Cyrillic script, but is not suited for logogram characters such as Chinese script and Japanese Kanji. A simple interface will also be provided for end users to link in commercial handwriting recognition engines.

VI. CONCLUSION

The new software was originally designed to cater for the research needs of MyAgeing, and has been shown to be at least as accurate as manual data entry, without the cost of existing commercial solutions. It is hoped that the public release of the source code will enable other smaller departments and groups which conduct survey research and clinical studies to derive the same benefits as our center.

Acknowledgment

The research on which this article is based was conducted as part fulfilment of a MSc(Gerontechnology) thesis by S.C.L. in 2016 at MyAgeing, Universiti Putra Malaysia.

REFERENCES

[1] S. C. Loke, "A software application for survey form design and processing for scientific use," M.S. thesis, MyAgeing., Univ. Putra Malaysia, Serdang, Malaysia, 2017.

[2] Kodak Alaris. (May 18, 2017). *Document Scanners*. [Online]. Available: https://www.kodakalaris.com/b2b/solutions/document-scanners

[3] S. C. Loke, K. F. Rahim, R. Kanesvaran, and T. W. Wong, "A prospective cohort study on the effect of various risk factors on hypoglycaemia in diabetics who fast during Ramadan," *Med. J. Malaysia*, vol. 65, pp. 3–6, Mar. 2010.

[4] S. C. Loke, R. Kanesvaran, R. Yahya, L. Fisal, T. W. Wong, and Y. Y. Loong, "Efficacy of an intravenous calcium gluconate infusion in controlling serum calcium after parathyroidectomy for secondary hyperparathyroidism," *Ann. Acad. Med. Singapore*, vol. 38, pp. 1074–1080, Dec. 2009.

[5] S. C. Loke, A. W. K. Tan, R. Dalan, and M. K.-S. Leow, "Pre-operative serum alkaline phosphatase as a predictor for hypocalcemia post-parathyroid adenectomy," *Int. J. Med. Sci.*, vol. 9, no. 7, pp. 611–616, 2012.

[6] C. Jinks, K. Jordan, and P. Croft, "Evaluation of a computer-assisted data entry procedure (including Teleform) for large-scale mailed surveys," *Comput. Biol. Med.*, vol. 33, no. 5, pp. 425–437, Sep. 2003.

[7] S. C. Loke, S. S. Abdullah, S. T. Chai, T. A. Hamid, and N. Yahaya, "Assessment of factors influencing morale in the elderly," *PLoS ONE*, vol. 6, no. 1, p. e16490, 2011.

[8] T. A. Hamid, S. Krishnaswamy, S. S. Abdullah, and Y. A. Momtaz, "Sociodemographic risk factors and correlates of dementia in older Malaysians," *Dement Geriatric Cognit. Disorders*, vol. 30, no. 6, pp. 533–539, 2010.

[9] S. C. Loke, W. S. Lim, Y. Someya, T. A. Hamid, and S. S. H. Nudin, "Examining the disability model from the international classification of functioning, disability, and health using a large data set of community-dwelling Malaysian older adults," *J. Aging Health*, vol. 28, no. 4, pp. 704–725, Oct. 2015.

[10] T. M. Jenkins *et al.*, "Evaluation of a Teleform-based data collection system: A multi-center obesity research case study," *Comput. Biol. Med.*, vol. 49, pp. 15–18, Jun. 2014.

[11] H. Quan, P. D. Biondo, C. Stiles, D. E. Moulin, and N. A. Hagen, "A patient-completed and optically read data acquisition system for clinical trials," *Contemp. Clin. Trials*, vol. 32, pp. 173–177, Mar. 2011.

[12] Hewlett-Packard Development Company. (May 18, 2017). *Teleform*. [Online]. Available: http://engage.opentext.com/products/teleform

[13] *GS1 General Specifications*, GS1, Brussels, Belgium, 2016.

[14] S. W. Singer and C. L. Meinert, "Format-independent data collection forms," *Control. Clin. Trials*, vol. 16, pp. 363–376, Dec. 1995.

[15] EMC Captiva. (Oct. 9, 2015). *Captive Embeddable Capture Technologies*. [Online]. Available: http://www.emc.com/enterprise-content-management/captiva/isis-drivers.htm

[16] S. C. Loke, "De-skewing of scanned documents using a new type of registration mark," to be published.

[17] S. C. Loke, "A new method of mark detection for software-based optical mark recognition," to be published.

[18] Z. Hu, J. Lin, and L. Wu, "Research on OCR post-processing applications for handwritten recognition based on analysis of scientific materials," in *Advances in Computer Science, Intelligent System and Environment*, D. Jin and S. Lin, Eds. Berlin, Germany: Springer, 2011, pp. 131–135.

[19] J. D. Neaton, A. G. Duchene, K. H. Svendsen, and D. Wentworth, "An examination of the efficiency of some quality assurance methods commonly employed in clinical trials," *Statist. Med.*, vol. 9, pp. 115–124, Jan./Feb. 1990.

[20] J. H. Tan, H. C. L. Tan, S. C. Loke, and S. A. Arulanantham, "Novel calcium infusion regimen after parathyroidectomy for renal hyperparathyroidism," *Nephrology*, vol. 22, pp. 308–315, Apr. 2017.

[21] J. Cohen, *Statistical Power Analysis for the Behavioral Sciences*. Hillsdale, NJ, USA: Lawrence Erlbaum Associates, 1988.

[22] F. Faul, E. Erdfelder, A. Buchner, and A.-G. Lang, "Statistical power analyses using G*Power 3.1: Tests for correlation and regression analyses," *Behavior Res. Methods*, vol. 41, pp. 1149–1160, Nov. 2009.

[23] Society for Clinical Data Management. (2015). *Good Clinical Data Management Practices (GCDMP)*. [Online]. Available: http://www.scdm.org/sitecore/content/be-bruga/scdm/Publications/gcdmp.aspx

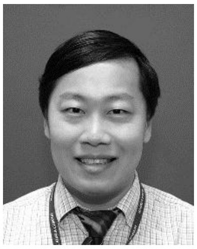

SENG CHEONG LOKE received the M.B.B.S. degree from the University of Melbourne Medical School, the M.R.C.P. degree from the Royal College of Physicians, U.K., and the F.A.M.S. degree in endocrinology from the Academy of Medicine, Singapore.

He was the Deputy Director of the National Gerontology Research Institute of Malaysia. He is currently a Consultant Physician and Endocrinologist with Serdang Hospital and Kuala Lumpur General Hospital, and an Associate Professor with Universiti Putra Malaysia. He has a strong research interest in both endocrinology and gerontology. He is investigating new ways in which information technology can transform traditional methods of research and patient care.

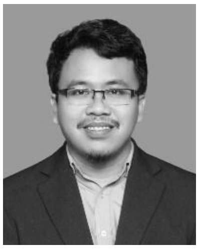

KHAIRUL AZHAR KASMIRAN received the Ph.D. degree from The University of Sydney, Australia, in 2012. He is currently a Senior Lecturer with the Department of Computer Science, Faculty of Computer Science and Information Technology, Universiti Putra Malaysia, Malaysia. His interests include information management, big data, text mining, performance engineering, and software development.

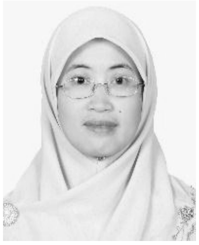

SHARIFAH AZIZAH HARON received the B.Economics degree (Hons.) from the International Islamic University, Malaysia, the M.Sc. degree in consumer economics from The University of Alabama, Tuscaloosa, and the Ph.D. degree in consumer and family economics from the University of Missouri, Columbia. She specializes in consumer and family economics, consumption economics, household economic wellbeing and poverty, and aging and intergenerational economics. She is currently an Associate Professor with the Universiti Putra Malaysia, and is currently serving as the Head of the Social Gerontology Laboratory, Malaysian Research Institute on Ageing.
